# Junction-mediating and regulatory protein (JMY) is a promoting protein for radial migration of cortical neurons

**DOI:** 10.1038/s41420-026-02974-7

**Published:** 2026-02-26

**Authors:** Xiang-ren Chen, Zhi-yi Chen, Shu-ya Qi, Qing-yun Huang, Li-hang Wei, Ming-yue Chen, Hao Li, Na Huang, Yong-xin Kang, Zhong-xin Guo, Xue-chao Jing, Guo-he Tan, Yuan-yuan Liu

**Affiliations:** 1 Guangxi Key Laboratory of Brain Science and Institute of Neuroscience, Key Laboratory of Human Development and Disease Research (Guangxi Medical University) by Education Department of Guangxi Zhuang Autonomous Region, Department of Human Anatomy, School of Basic Medical Sciences, Guangxi Medical University, Nanning, Guangxi China; 2 Department of Neurology, The First Affiliated Hospital of Guangxi Medical University, Nanning, Guangxi China; 3 Department of Neurosurgery, The First Affiliated Hospital of Guangxi Medical University, Nanning, Guangxi China; 4 Key Laboratory of Longevity and Aging-Related Diseases of the Chinese Ministry of Education, Nanning, Guangxi China; 5 Guangxi Key Laboratory of Regenerative Medicine, Collaborative Innovation Centre of Regenerative Medicine and Medical BioResource Development and Application Co-constructed by the Province and Ministry, Nanning, Guangxi China; 6 China-ASEAN Research Center for Innovation and Development in Brain Science, Nanning, Guangxi China

**Keywords:** Neuronal development, Developmental neurogenesis

## Abstract

Radial neuronal migration is a critical process for the formation of the cerebral cortical layers. Although transcriptional regulators are implicated in neuronal migration, the molecular mechanisms remain incompletely understood. Junction-mediating and regulatory protein (JMY), a p53 coactivator with established roles in embryonic development, has an unclear role in neurodevelopment. Here, we found that JMY is highly expressed in the developing mouse brain, particularly in the ventricular zone and subventricular zone, regions associated with neurogenesis. Knockdown of *Jmy* resulted in delayed radial migration of cortical neurons, disrupted cell cycle exit, impaired neuronal differentiation, reduced dendritic complexity and produced laminar disorganization. Behavioral tests showed spatial learning and memory deficits in JMY-deficient mice. Proteomic analysis suggested that knocking out *Jmy* in the mouse brain affects cell cycle-related pathways. Our findings indicate an important role of JMY in neural development and cognitive function in the developing mouse brain, providing novel insights into the molecular mechanisms underlying neuronal migration during corticogenesis.

## Introduction

Cerebral cortical development is the basis for brain structure and function, relying on tightly regulated processes including neural stem cell proliferation, differentiation, migration and neuronal localization [1, 2]. Neuronal migration is essential for cerebral cortical development, ensuring that postmitotic neurons move from their proliferative zones to designated cortical layers, thereby forming the six-layered neocortex structure that underlies higher-order cognitive functions [3]. Disruptions of this process can lead to severe developmental abnormalities and cognitive dysfunctions, including intellectual disabilities, epilepsy, severe learning disabilities and autism spectrum disorders [4, 5]. The migration of neural progenitor cells and their differentiation into specific neuronal subtypes is a critical process for normal brain development. These processes are orchestrated by a wide array of signaling molecules and pathways that regulate cell cycle progression, cytoskeletal reorganization, and cell fate determination [6].

Among these, the Wiskott‒Aldrich Syndrome Protein (WASP) family of proteins is critical in regulating various cellular processes, including actin polymerization, cell migration, and differentiation [7, 8]. Junction-mediating and regulatory protein (JMY) is a member of the WASP family [9], which was initially identified as a transcriptional coactivator of p53 [10]. Studies have shown that JMY acts as a coactivator of p53, thereby increasing the transcriptional activity of p53 target genes involved in cell cycle regulation and apoptosis [9, 11]. Activation of p53 is crucial for maintaining proper neural development, influencing the balance between the self-renewal and differentiation of neural progenitor cells [12, 13], and ensuring the correct timing of neuronal maturation [14, 15]. P53 activates several downstream target genes that are involved in cell cycle arrest, DNA repair, and apoptosis, such as *p21* and *Gadd45α* [16, 17]. These proteins are essential for regulating cell cycle checkpoints and preserving genomic integrity, processes that are required for proper neuronal maturation and cortical organization [16, 18].

Given the importance of p53 in neural development, JMY is likely to contribute to cortical neuron development and cognitive functions. It has been reported that JMY plays crucial roles in mammalian oocyte maturation in both mice and pigs [19, 20]. Furthermore, knockdown of *Jmy* in porcine embryos caused developmental abnormalities in porcine and mouse embryos [21]. Thus, these studies suggest that JMY may also play an important role in neuronal development and function. However, the expression and function of JMY in the nervous system remain largely unexplored.

In this study, we found that *Jmy* is highly expressed in the brain during the early stage of development and is expressed in both immature and mature neurons. Reducing *Jmy* expression could impair the migration of cortical neurons. Moreover, JMY can cause progenitor cells to shift from proliferative cell division to differentiative cell division. In behavior tests, conditional *Jmy*-knockout mice exhibited impaired learning and memory. Our findings suggest that JMY has an important role in brain development and provide new evidence for a potential mechanism regulating cortical neuronal development.

## Results

### JMY is highly expressed in the developing mouse brain

**Fig. 1:**
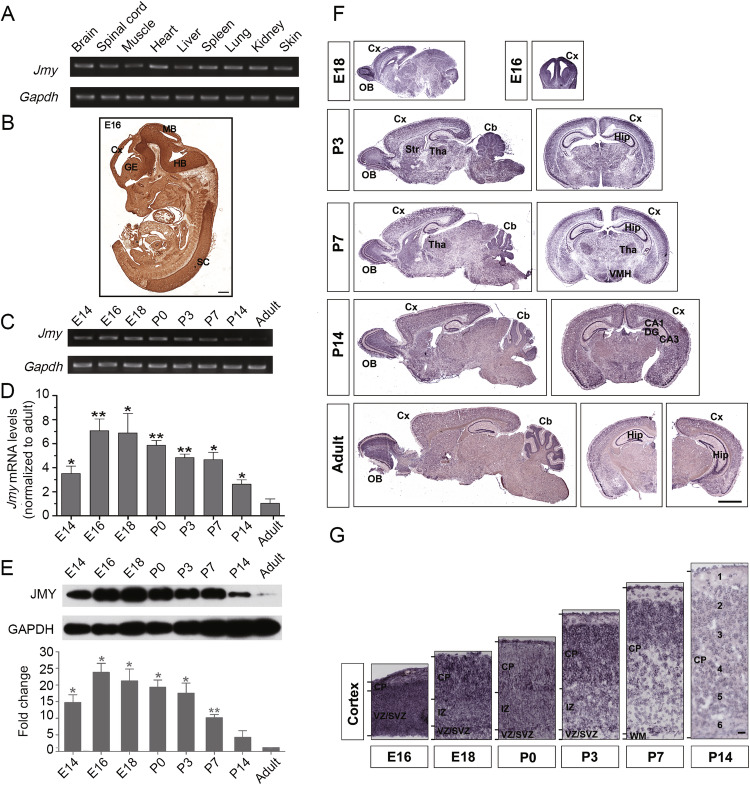
JMY is expressed at high levels in the developing mouse brain. **A** RT‒PCR analysis of *Jmy* mRNA levels in the indicated tissues from different organs of P7 mice. **B** DAB staining showing the expression of the JMY protein in a sagittal section of an E16 mouse embryo. Cx, cortex; GE, ganglionic eminence; MB, midbrain; HB, hindbrain; SC, spinal cord. Scale bar, 300 μm. **C** RT‒PCR assays showing the temporal distribution of *Jmy* expression in whole-brain lysates from mice at the indicated stages of development. GAPDH was used as an internal control. **D** Quantification of *Jmy* mRNA levels in brain lysates (*n* = 6 for each timepoint, *p < 0.05, **p < 0.01, mean ± SEM) via quantitative real-time PCR, with normalization to the expression levels in the adult brain. **E** Western blot showing the protein expression of JMY in the mouse brain at the indicated developmental stages. The results of the statistical analysis are shown at the bottom. The data were normalized to the expression levels in the adult brain. *n* = 3, *p < 0.05, **p < 0.01; the data represent the means ± SEM. **F** In situ hybridization showing the temporal expression of *Jmy* mRNA in the mouse brain at different developmental stages. Cx Cortex, Cb Cerebellum, Hip Hippocampus, OB Olfactory Bulb, Tha Thalamus, DG Dentate Gyrus, Str Striatum. A high-magnification image of the cortex area is shown in (**G**). CP Cortical Plate, VZ Ventricular Zone, SVZ Subventricular Zone, IZ Intermediate Zone, WM White Matter. Scale bars, 2 mm (**F**) and 50 μm (**G**).

JMY is widely expressed in mammalian tissues and cell lines [22], and its function in peripheral organs and tissues has been preliminarily studied. However, the expression pattern of JMY in the nervous system during development remains unclear. Using RT‒PCR, we detected that *Jmy* mRNA was expressed in all examined tissues from P7 mice, with higher levels in the brain, heart, spleen, lung and kidney and lower in the spinal cord, muscle, liver and skin, which is consistent with previous findings [22] (Fig. 1A). Moreover, immunohistochemical analysis of embryonic day 16 (E16) mouse embryos, by using a commercial anti-JMY antibody, showed JMY is abundant in the cerebral cortex, ganglionic eminence, midbrain and hindbrain, with significantly higher levels in these neural tissues compared to other tissues (Fig. 1B). Considering the high-level expression of JMY in the nervous system of developing mice, we hypothesized that JMY might exhibit a specific temporal expression pattern during brain development. Indeed, *Jmy* mRNA was detected in the mouse cerebral cortex throughout development from E14 to adulthood, with peak expression around E18. Postnatal expression gradually decreased, especially after P14, reaching minimal levels in adulthood (Fig. 1C). Real-time PCR analysis also showed this temporal expression pattern (Fig. 1D). Furthermore, we performed western blotting and found JMY protein levels were highest between E16-E18, approximately 20-25 times greater than in the adult brain (Fig. 1E). As the age of the mice increased, the protein expression level gradually decreased to its lowest level in adulthood, which is consistent with the above RT‒PCR results. Taken together, these results indicated transient high expression of JMY in the mouse brain during the early stage of development, suggesting that JMY may play a specific role in brain development.

To further investigate the spatial expression pattern of *Jmy* in the brain, we next performed in situ hybridization using a specific *Jmy* mRNA probe. Our analysis revealed that *Jmy* is predominantly expressed in neurogenic regions, including the VZ and SVZ at E16 (Fig. 1F), suggesting the potential roles of JMY in neuronal proliferation and differentiation. Importantly, JMY began to be expressed in mature regions of the cortex at E18, such as the cortical plate (CP), which was more obvious during the early postnatal period (P3–P7), suggesting that JMY is also expressed at high levels in differentiated neurons. At E18, JMY was expressed not only in progenitor-rich zones (VZ/SVZ) but also in the cortical plate (CP). This pattern highlights that JMY expression spans both the period of active neuronal migration and the subsequent maturation phase, with high and evenly distributed levels across cortical layers that gradually decrease after P7, when neuronal migration is largely complete (Fig. 1G). These results suggested that JMY may play a potential role in the division and differentiation of neural precursor cells, as well as in the early development of differentiated neurons.

**Fig. 2:**
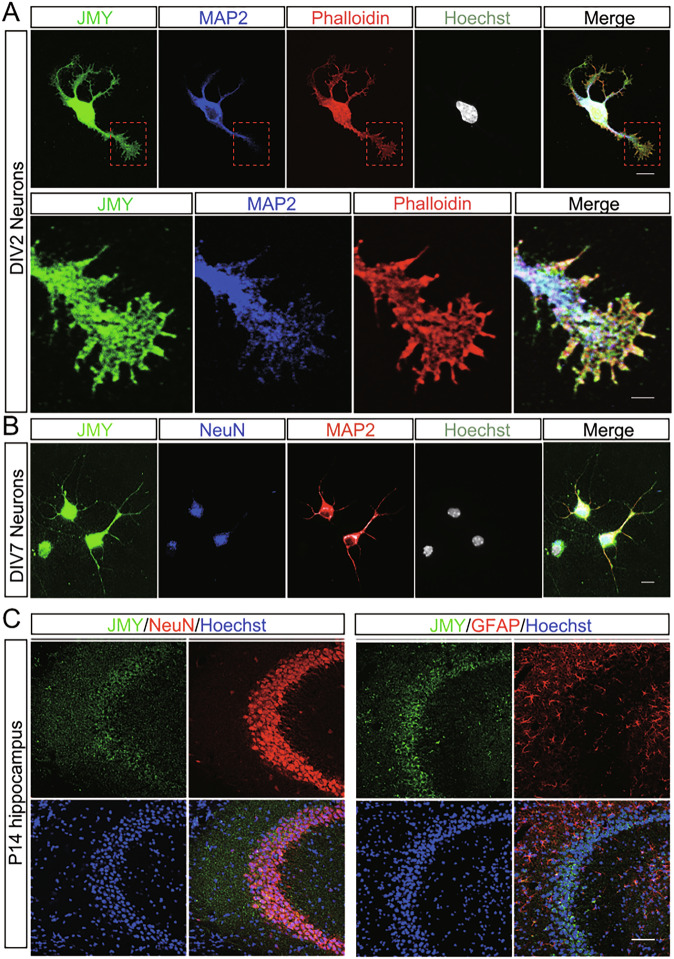
Expression pattern of JMY protein in developing neurons in vitro and in vivo. **A** Representative image of cultured cortical neurons costained with JMY and MAP2 antibodies. The subcellular location of endogenous JMY is shown in green. The lower panel shows a magnified growth cone from the upper panel. F-actin and the nucleus were visualized by Phalloidin (red) and Hoechst (gray) staining, respectively. Scale bars, 10 µm (upper panel) and 2 µm (lower panel). **B** JMY immunocytochemistry in DIV7 cultured cortical neurons. MAP2 and NeuN were used as markers for the cytosol and nucleus, respectively. Scale bar, 15 µm. **C** Immunostaining of brain slices from mice at P14. GFAP were used as markers of the astrocytes. Scale bar, 60 µm.

Moreover, we investigated the expression and subcellular localization of JMY in neurons. We cultured cortical neurons at different time points in vitro and performed immunocytochemical staining to explore the distribution of JMY in immature neurons. In 2 days in vitro (DIV2) neurons, we found that JMY colocalized with microtubules labeled by microtubule-associated protein 2 (MAP2) in the soma and dendrites, as well as with F-actin in the growth cones (Fig. 2A). These findings indicate that JMY is abundantly expressed in the soma, dendrites and growth cones of cultured neurons. We further observed that JMY co-localized with both the cytosol expressing MAP2 and nucleus expressing NeuN, which is consistent with previous reports [22] (Fig. 2B). In addition, JMY was expressed in NeuN-positive neurons, but not in GFAP-positive astrocytes, in P14 mouse hippocampus (Fig. 2C).

These above findings suggested that JMY is widely expressed in the brain, especially during early cortical development, in both immature and mature neurons, which indicates that it may be involved in neuronal proliferation, differentiation, and migration.

### JMY promotes radial migration of cortical neurons during early embryonic development

**Fig. 3:**
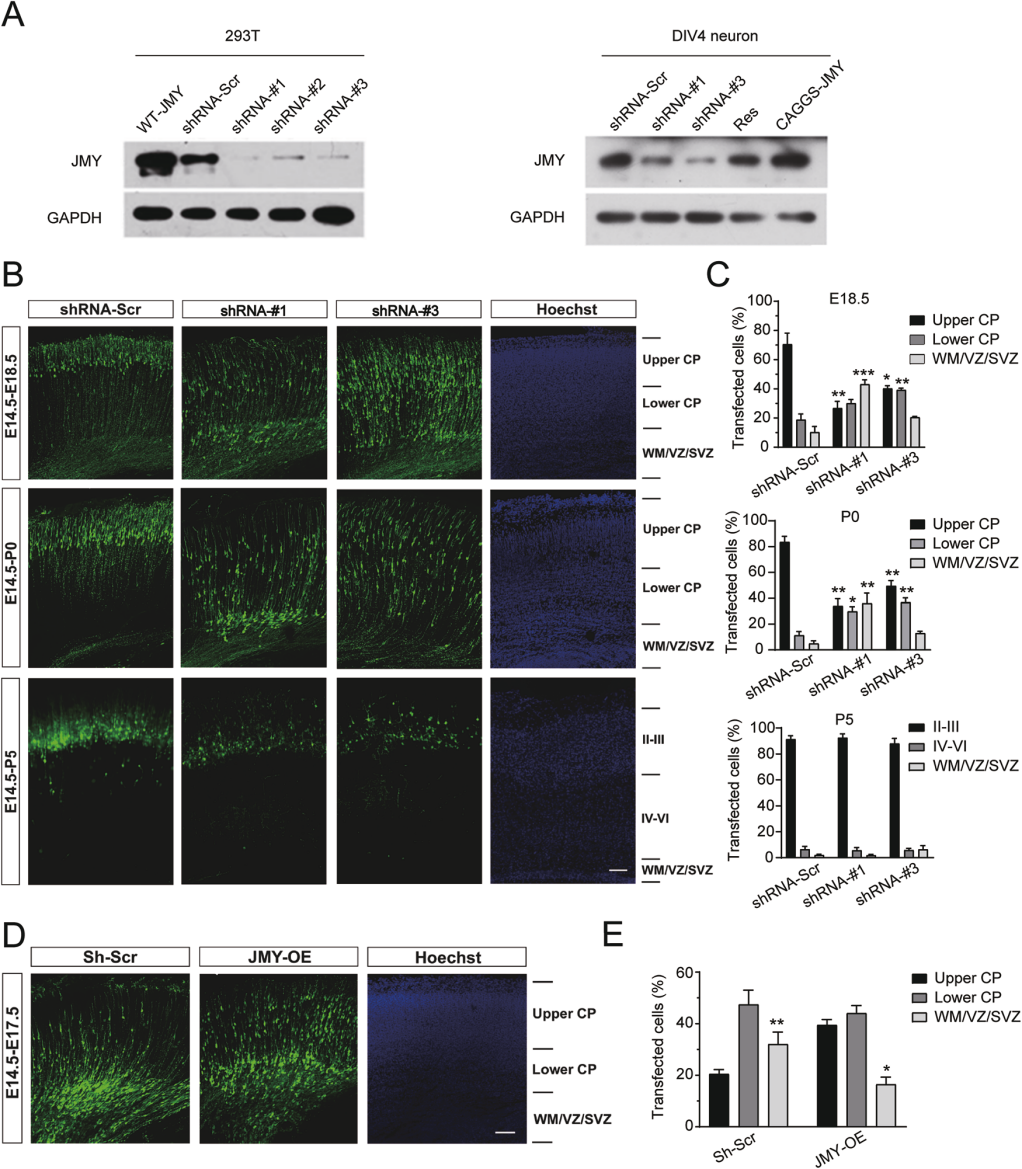
Downregulation of *Jmy* expression impairs the radial migration of cortical neurons in vivo. **A** 293T cells were transfected with a wild-type JMY expression plasmid (wt-JMY), scrambled shRNA (shRNA-Scr), or three independent JMY-targeting shRNAs (shRNA-#1, shRNA-#2, and shRNA-#3). Dissociated DIV4 primary neurons were infected with scrambled shRNA (shRNA-Scr), shRNA-#1, or shRNA-#3, or with an shRNA-resistant JMY rescue construct (Res). An additional condition involved expressing JMY from a CAGGS-JMY overexpression plasmid (CAGGS-JMY). Western blot analysis was performed to assess JMY protein levels in both 293 T cells and DIV4 neurons following knockdown and rescue. GAPDH was used as an internal loading control. **B** Coronal sections of brains from E18.5–P5 mice electroporated at E14.5 with EGFP plus the shRNA-Scr (left panel), shRNA-#1 (left-middle panel), and shRNA-#3 (right-middle panel) constructs, respectively. Cells derived from transfected cortical progenitor cells are shown in green. Sections were counterstained with Hoechst (blue; right panel), showing stained neighboring sections of the corresponding cortex. The cortical layers are indicated on the right. CP cortical plate, WM white matter, VZ ventricular zone, SVZ subventricular zone. Scale bar, 60 μm. **C** Analysis of the distribution of transfected cortical neurons in electroporated brains across different cortical zones at the indicated developmental stages. The results are presented as the mean ± SEM. (*n* = 3, *p < 0.05, **p < 0.01, ***p < 0.001). Statistical significance was assessed by comparison with the shRNA-Scr group. **D** Coronal sections of brains from E18.5 mice electroporated at E14.5 with EGFP plus shRNA-Scr (Sh-Scr) or full-length *Jmy* (JMY-OE), respectively. Cells derived from transfected cortical progenitor cells are shown in green. Sections were counterstained with Hoechst (blue; right panel), showing stained neighboring sections of the corresponding cortex. The cortical layers are indicated on the right. CP cortical plate, WM white matter, VZ ventricular zone, SVZ subventricular zone. Scale bar, 60 μm. **E** Analysis of the distribution of transfected cortical neurons in electroporated brains across different cortical zones at the indicated developmental stages. The results are presented as the means ± SEM. (*n* = 3, *p < 0.05, **p < 0.01, ***p < 0.001). Statistical significance was assessed by comparison with the shRNA-Scr group.

Given the prominent expression of JMY in neurogenic regions during embryonic stages, we next investigated its functional role in cortical neuron migration using in utero electroporation. We co-injected GFP-expressing plasmids with either JMY-targeting shRNAs (shRNA-#1, shRNA-#3) or scrambled control (shRNA-Scr), as well as a full-length *Jmy* overexpression construct [23], into E14.5 embryonic mouse lateral ventricles (Fig. 3A). Under an appropriate electric field, the plasmid DNA was transfected into the cells close to the ventricle of the cerebral cortex in fetal mice [24]. Immunohistochemistry and morphological assessments were carried out at E18.5, P0, and P5. JMY knockdown with shRNA-#1 resulted in significant retention of GFP-positive cells in the VZ/SVZ, with few neurons reaching the intermediate zone (IZ) and cortical plate (CP) (Fig. 3B, C). Meanwhile, control mice (shRNA-Scr) showed efficient migration to the CP with minimal SVZ retention (Fig. 3B, C). Knocking down *Jmy* with shRNA-#3 also caused a significant reduction in migration compared with that in the control group, although to a lesser extent than observed in the shRNA-#1 group (Fig. 3B, C). This phenomenon persisted until after birth, and the number of GFP-positive cells that reached the upper CP was comparable between the shRNA construct and scrambled control groups at P5 (Fig. 3B, C). These findings indicate that reduced JMY expression in the embryonic brain could cause a delay in the radial migration of cortical neurons during early embryonic development, but this phenotype normalized during postnatal development. To further confirm the role of JMY in neuronal migration in the developing cortex, we also performed an overexpression experiment in which full-length *Jmy* and GFP plasmids were co-transfected into the developing mouse cortex, and the results revealed that JMY overexpression enhanced neuronal migration to the CP while reducing VZ/SVZ retention (Fig. 3D, E). These results indicate that JMY can promote radial migration of cortical neurons during embryonic development.

### JMY promotes neural progenitor cell cycle exit and differentiation in the developing cerebral cortex

**Fig. 4:**
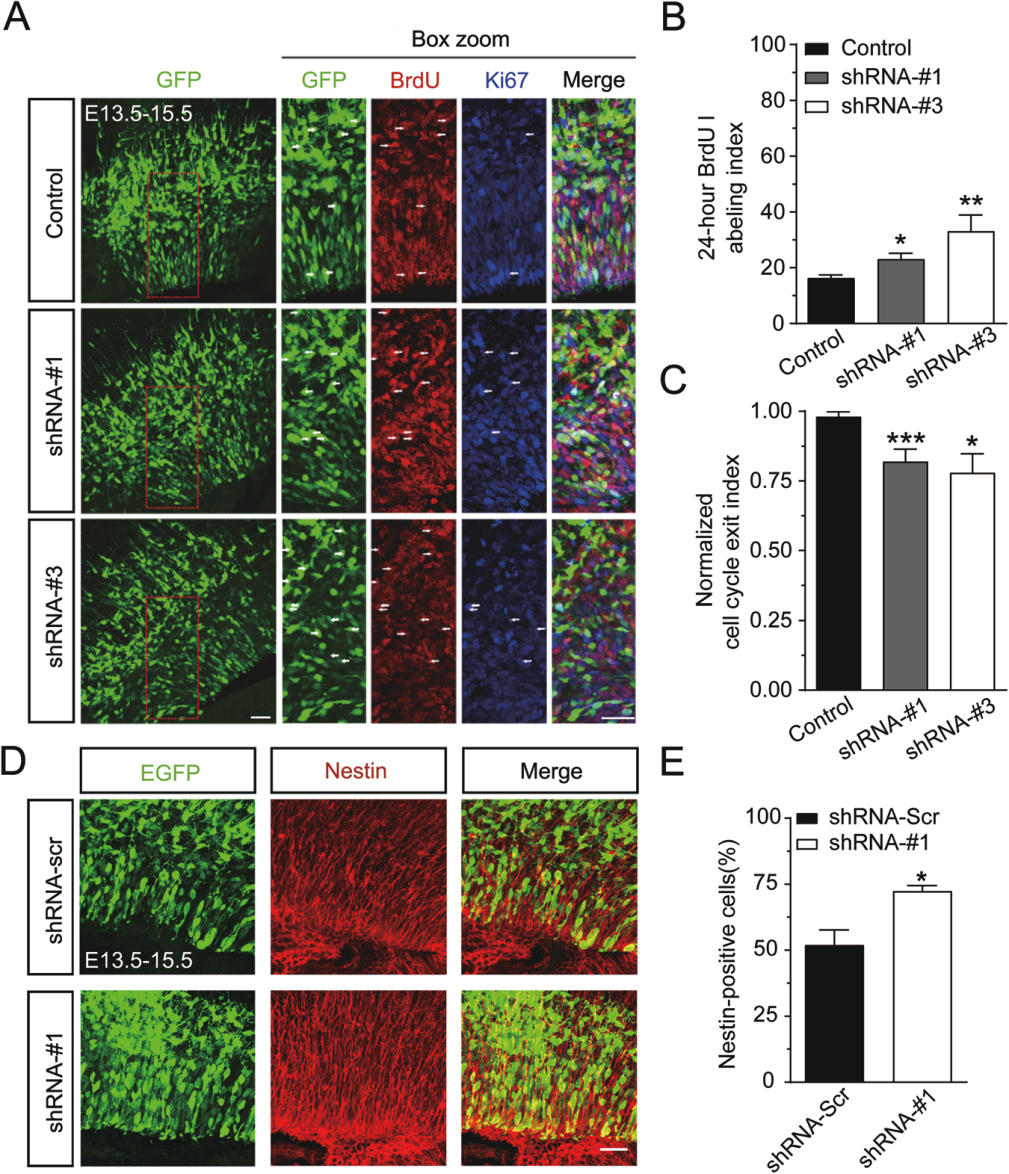
JMY regulates progenitor cell proliferation differentiation in utero. **A** Representative images showing BrdU and Ki67 incorporation in the mouse brain subjected to JMY knockdown. The mouse brains electroporated at E13.5 were pulse labeled with BrdU for 2 h before the mice were sacrificed at E15.5. **B** Quantitative analysis of BrdU-labeled cells in electroporated mouse brains. The bar graph shows the percentage of BrdU and GFP double-positive cells among total GFP-positive cells in the VZ/SVZ. **C** The cell cycle exit index was calculated as the ratio of BrdU^+^Ki67^−^ GFP^+^ cells to the total population of BrdU^+^GFP^+^ cells (*n* = 4, *p < 0.05, **p < 0.01, ***p < 0.001). Scale bar, 20 μm. **D** Representative images of Nestin-positive cells in the mouse brain subjected to JMY knockdown. The mouse brain electroporated at E13.5 were pulse labeled with BrdU for 2 h before the mice were sacrificed at E15.5. **E** Quantitative analysis of Nestin-positive cells among GFP-positive neurons. The bar graph shows the percentage of Nestin and GFP double-positive cells among total GFP-positive cells in the VZ/SVZ (*n* = 3, *p < 0.05). Scale bar, 20 μm. All the data are presented as the mean ± SEM.

Previous studies revealed that early JMY is distributed mainly in the VZ/SVZ of the embryonic brain as a nuclear p53 cofactor. Therefore, we hypothesized it might regulate neuronal division and proliferation. To verify this, we used BrdU and Ki67 labeling in electroporated brains to assess cell proliferation and cell cycle exit in the VZ/SVZ [25–27]. The cell cycle exit index was calculated as the ratio of BrdU^+^Ki67^−^GFP^+^ cells to the total population of BrdU^+^GFP^+^ cells to analyze cell cycle kinetics in the VZ of the cerebral cortex (Fig. 4A).

The results revealed that JMY knockdown significantly increased BrdU-positive cells in the VZ (Fig. 4B) and reduced the cell cycle exit index (Fig. 4C), suggesting that JMY can cause progenitor cells to shift from proliferative cell division to differentiation.

To further confirm the ability of JMY in regulating cell proliferation and differentiation, we conducted immunohistochemistry using a Nestin antibody in the developing mouse brain following co-transfection with either GFP plus shRNA-scr plasmids or the shRNA-#1 plasmids (Fig. 4D). Nestin is a neural stem/progenitor cell marker [28, 29], and its expression declines as progenitors differentiate into neurons or glia [30, 31]. During early development, proliferative divisions expand the progenitor pool, whereas later differentiative divisions reduce it. Therefore, it is possible to investigate the rate of proliferation and the fraction of neural progenitor cells that exit the cell cycle by observing the size of the remaining progenitor pool. Immunostaining revealed that the percentage of Nestin and GFP double-positive cells among total GFP-positive cells was greater in the VZ/SVZ in the brains of mice subjected to JMY knockdown via RNAi than in those of the control mice treated with shRNA-scr (Fig. 4E). These results suggest that JMY promotes differentiative divisions at the expense of proliferative divisions.

### JMY enhances neuronal morphology during cortical development

**Fig. 5:**
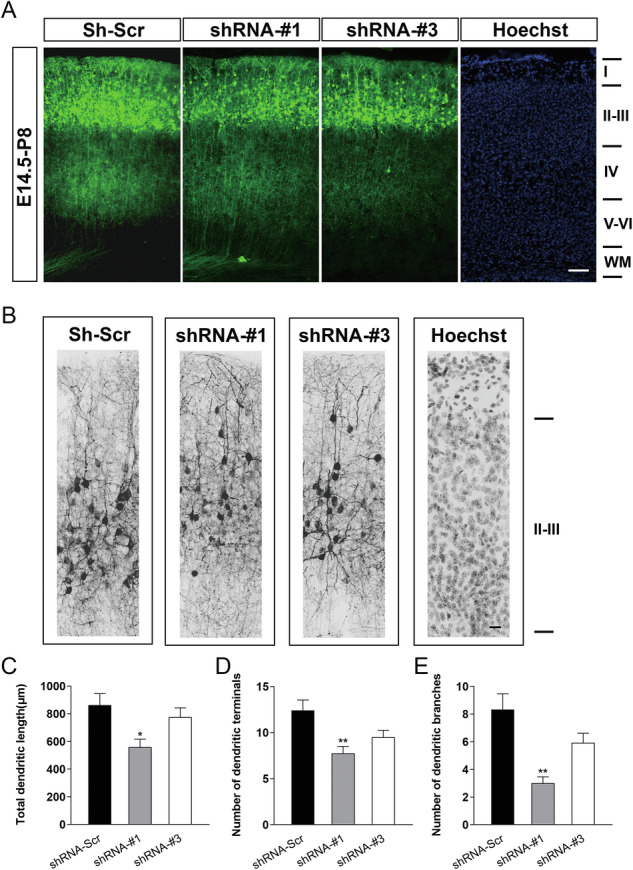
Knockdown of JMY impairs cortical neuron migration and dendritic morphogenesis in vivo. **A** In utero electroporation of GFP-expressing scrambled shRNA (Sh-Scr) or *Jmy*-shRNAs (#1 and #3) was performed at E14.5, and the brains were analyzed at P8. Cortical neurons transfected with Sh-Scr migrated properly into layers II–III, whereas JMY knockdown led to delayed migration, with many neurons retained in deeper layers. Hoechst staining indicates cortical lamination. Scale bar, 50 μm. **B** Representative images of GFP^+^ neurons within layer II–III cortex revealed simplified dendritic morphology in JMY-deficient neurons, compared with controls. Inverted gray-scale images are shown for better visualization of fine processes. Scale bar, 20 μm. **C–E** Quantification of dendritic parameters. JMY knockdown significantly reduced total dendritic length (**C**), the number of dendritic terminals (**D**), and the number of dendritic branches (**E**). *n* = 6 for each group, *p < 0.05, **p < 0.01, mean ± SEM.

To further investigate the role of JMY in neuronal development, following our previous observations on its impact on neuronal migration, proliferation, and differentiation, we next assessed its effect on neuronal morphogenesis during cortical development. In utero electroporation of *Jmy*-shRNA plasmids at E14.5, followed by analysis of GFP^+^ neurons at P8, showed that control neurons migrated efficiently to the upper cortical layers and developed elaborate dendritic arbors (Fig. 5A). In contrast, JMY-deficient neurons exhibited delayed migration and simplified dendritic morphology, with reduced total dendritic length, fewer dendritic terminals, and decreased branching complexity (Fig. 5B–E). Together, these observations indicate that JMY knockdown significantly impairs dendritic development during cortical maturation.

### JMY regulates cortical laminar organization in adult mice

**Fig. 6:**
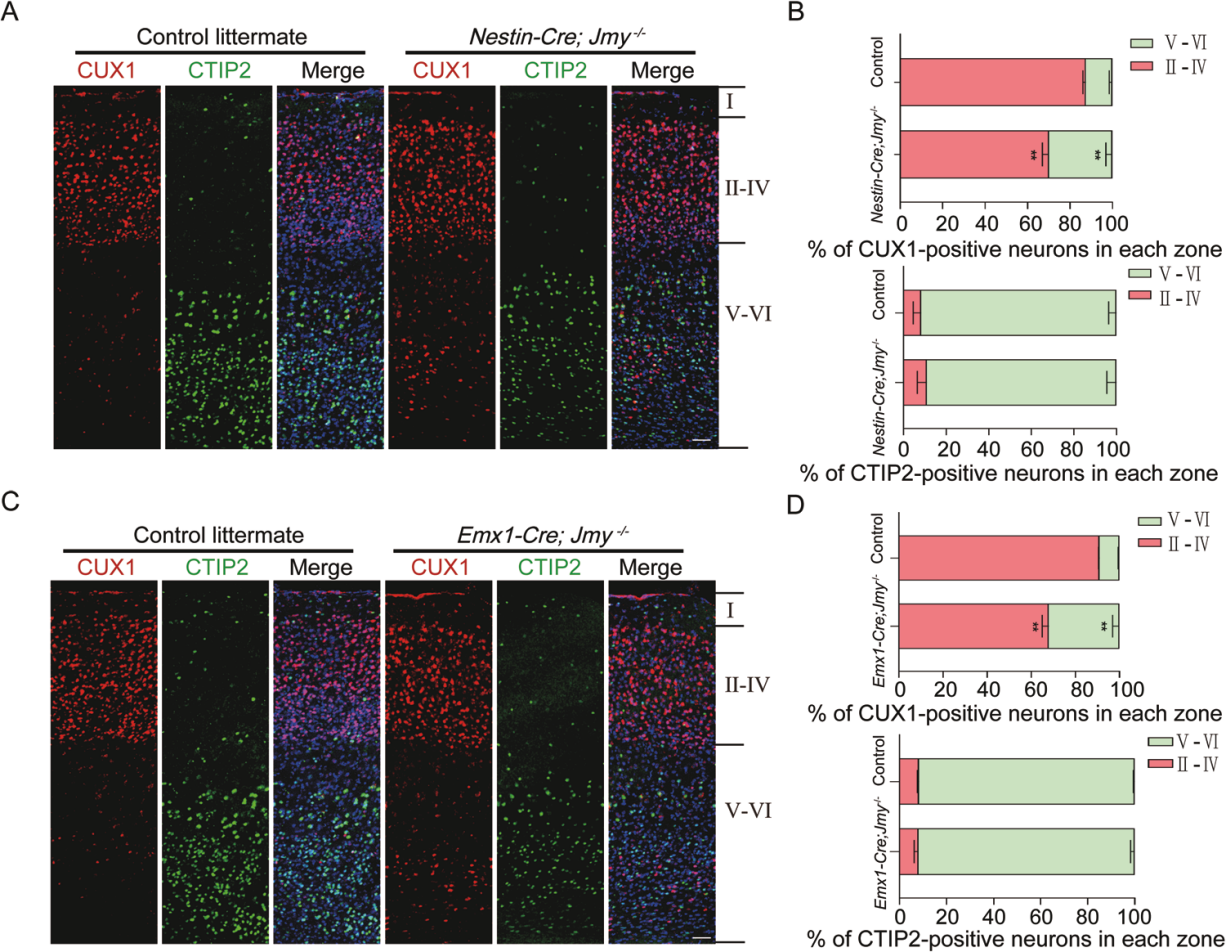
Cortical laminar disorganization in adult *Jmy* conditional knockout mice. **A** Coronal sections of the adult neocortex from *Nestin-Cre;Jmy*^*−/−*^ mice stained for CUX1 (red), CTIP2 (green), and hoechst (blue). Scale bar, 20 μm. **B** Quantification of the laminar distribution of CUX1-positive and CTIP2-positive neurons in control littermates and *Nestin-Cre;Jmy*^*−/−*^ mice. *n* = 4 for each group, **p < 0.01 versus control, mean ± SEM. **C** Coronal sections of the adult neocortex from *Emx1-Cre;Jmy*^*−/−*^ mice stained for the same markers. Scale bar, 20 μm. **D** Quantification of the laminar distribution of CUX1-positive and CTIP2-positive neurons in control littermates and *Emx1-Cre;Jmy*^*−/−*^ mice. *n* = 4 for each group, **p < 0.01 versus control, mean ± SEM.

To investigate the role of JMY in cortical development, we generated conditional knockout mice using *Nestin-Cre* drivers in which JMY was specifically deleted in neural progenitor cells (Fig. S1A, B). Efficient deletion of JMY was confirmed in both lines by RT–PCR and Western blot of cortical lysates (Fig. S1C, D). While Nissl staining showed no gross abnormalities in overall cortical or hippocampal size or cytoarchitecture (Fig. S1E), cortical lamination analysis showed significant disorganization of the upper cortical layers. Immunostaining for CUX1 (upper layers II–IV), and CTIP2 (deep layers V–VI) revealed that a proportion of CUX1^+^ neurons failed to reach the superficial layers and remained ectopically located in deeper cortical zones in *Nestin-Cre;Jmy*^*−/−*^ mice, indicating disrupted radial migration and impaired cortical organization. In contrast, the distribution of CTIP2^+^ neurons was largely comparable to controls (Fig. 6A, B).

Similar results were obtained with *Emx1-Cre;Jmy*^*−/−*^ mice, where *Jmy* deletion is restricted to the cortex and hippocampus (Fig. S1F–J). These mice also exhibited disrupted lamination with mislocalized CUX1^+^ neurons, whereas CTIP2^+^ neuronal positioning remained unaffected (Fig. 6C, D). These findings further confirm the role of JMY in regulating neuronal migration and cortical lamination.

### Spatial learning and memory are impaired in JMY-deficient mice

**Fig. 7:**
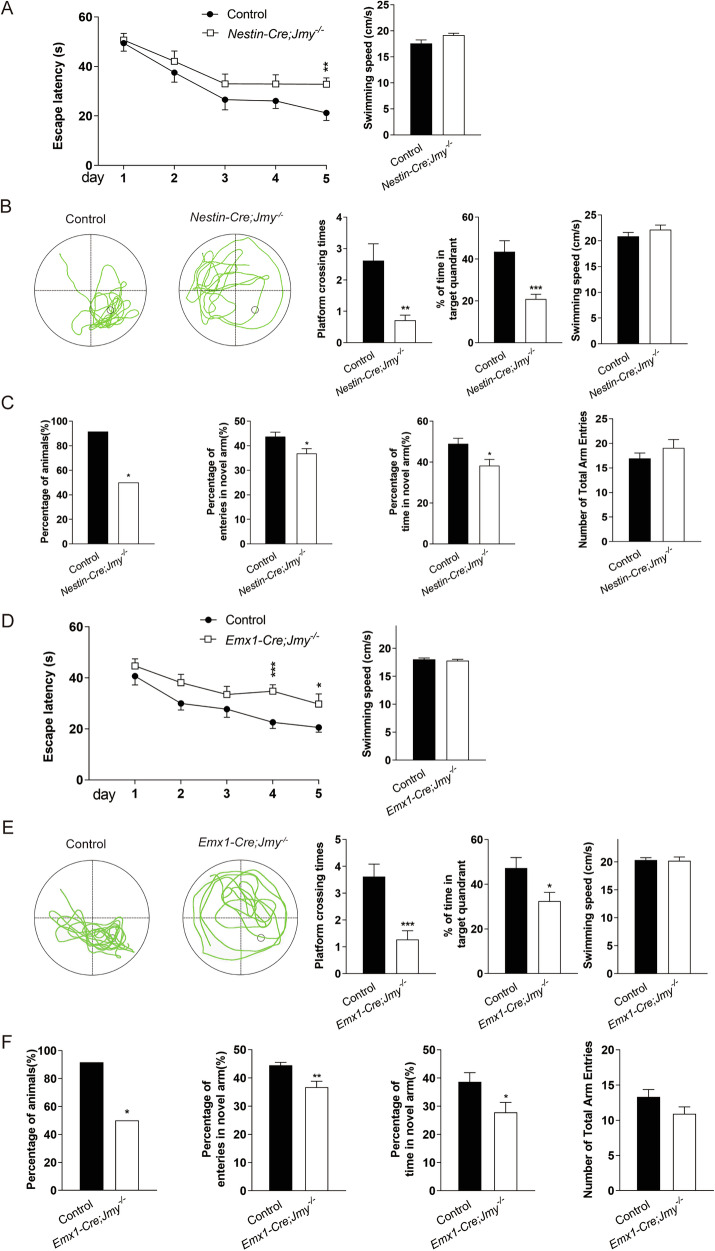
Spatial learning and memory are impaired in *Nestin-Cre;Jmy*^*−/−*^ and *Emx1-Cre;Jmy*^*−/−*^ mice. **A** The results of the Morris water maze test revealed that the escape latency of *Nestin-Cre;Jmy*^*−/−*^ mice (*n* = 14) was prolonged during the 5-day training period compared with that of their control littermates (*n* = 13). The swimming speed of *Nestin-Cre;Jmy*^*−/−*^ mice did not appear to change during the 5-day training period**. B** Representative search paths for mice of each group in the probe trial. The spatial memory of the control littermates and *Nestin-Cre;Jmy*^*−/−*^ mice is indicated by the number of platform crossings after training and the time spent in the quadrant with the hidden platform removed. The swimming speed of *Nestin-Cre;Jmy*^*−/−*^ mice did not appear to change on the 6^th^ day when the hidden platform was removed**. C** Impaired learning and memory in *Nestin-Cre;Jmy*^*−/−*^ mice, as measured with the Y-maze. The percentage of animals selecting the novel arm as the first choice is shown (left), and the number of arm entries (middle) and dwelling time (right) of *Nestin-Cre;Jmy*^*−/−*^ mice (*n* = 12) and their control littermates (*n* = 12) in the Y-maze 1 h after the first encounter with the partially opened maze are shown. **D** The Morris water maze test revealed that the escape latency of *Emx1-Cre;Jmy*^*−/−*^ mice (*n* = 18) was prolonged during the 5-day training period, compared with that of their control littermates (*n* = 18). The swimming speed of *Emx1-Cre;Jmy*^*−/−*^ mice did not appear to change during the 5-day training period. **E** Representative search paths for mice of each group in the probe trial. The spatial memory of the control littermate and *Emx1-Cre;Jmy*^*−/−*^ mice is indicated by the number of platform crossings after training and the time spent in the quadrant with the hidden platform removed. The swimming speed of *Emx1-Cre;Jmy*^*−/−*^ mice did not appear to change on the 6^th^ day when the hidden platform was removed. **F** Impaired learning and memory in *Emx1-Cre;Jmy*^*−/−*^ mice, as measured with the Y-maze. The percentage of animals selecting the novel arm as the first choice is shown (Left), and the number of arm entries (middle) and the dwelling time (right) of *Emx1-Cre;Jmy*^*−/−*^ mice (*n* = 12) and their control littermates (*n* = 12) in the Y-maze 1 h after the first encounter with the partially opened maze are shown. *p < 0.05, **p < 0.01, ***p < 0.001.

Given the pronounced laminar disorganization observed in both *Nestin-Cre;Jmy*^*−/−*^ and *Emx1-Cre;Jmy*^*−/−*^ mice, we next evaluated whether these developmental abnormalities translate into deficits in higher-order functions dependent on cortical–hippocampal circuits. To probe this, we evaluated spatial learning and memory in *Jmy* cKO mice using established behavioral paradigms, including the Morris water maze and the Y-maze. First, the Morris water maze results revealed that, compared with control littermate mice, *Nestin-Cre;Jmy*^*−/−*^ mice exhibited an increased escaping latency during the training process (Fig. 7A). To further assess the retention of spatial memory in *Nestin-Cre;Jmy*^*−/−*^ mice, we evaluated their performance in the probe trials conducted thereafter (Fig. 7B). On the 6th day of training in the MWM test, the *Nestin-Cre;Jmy*^*−/−*^ mice presented significant decreases in the number of platform crossings and the time spent in the target quadrants in the probe trial phase (Fig. 7B). Taken together, the above results suggest that *Jmy* knockout can affect spatial memory retention in *Nestin-Cre;Jmy*^*−/−*^ mice. Regarding novel arm exploration in the Y-maze test, a deficit in spatial memory of *Nestin-Cre;Jmy*^*−/−*^ mice was evident when the arm chosen for the first entry was recorded (Fig. 7C). Moreover, littermate control mice more frequently entered the novel arm of the maze, which was previously unvisited. In contrast, *Nestin-Cre;Jmy*^*−/−*^ mice showed no preference toward the novel arm and entered randomly into various arms. Specifically, these cKO mice presented lower percentages of time spent in the novel arm, fewer entries into the novel arm, and a decreased duration of stay in the novel arm (Fig. 7C). Spatial memory and recognition memory are influenced by both hippocampal and cortical lesions [32, 33]. *Emx1-Cre;Jmy*^*−/−*^ mice showed similar impairments. In the Morris water maze test, these mice exhibited increased escape latencies during the training phase compared with their control littermates, indicating difficulty in learning (Fig. 7D). Additionally, in the probe trials conducted thereafter, the *Emx1-Cre;Jmy*^*−/−*^ mice presented a significant reduction in the number of platform crossings and decreased time spent in the target quadrants, suggesting impaired spatial memory retention (Fig. 7E). The Y-maze test further corroborated these findings, as the knockout mice displayed a lack of preference for the novel arm and randomly entered into various arms, unlike the littermate control mice, which predominantly explored the novel, previously unvisited arm (Fig. 7F). These results underscore the critical role of JMY in the structural and functional integrity of the cortex and hippocampus, which are essential for maintaining proper spatial memory and recognition.

### JMY deficiency disrupts cell cycle regulation

**Fig. 8:**
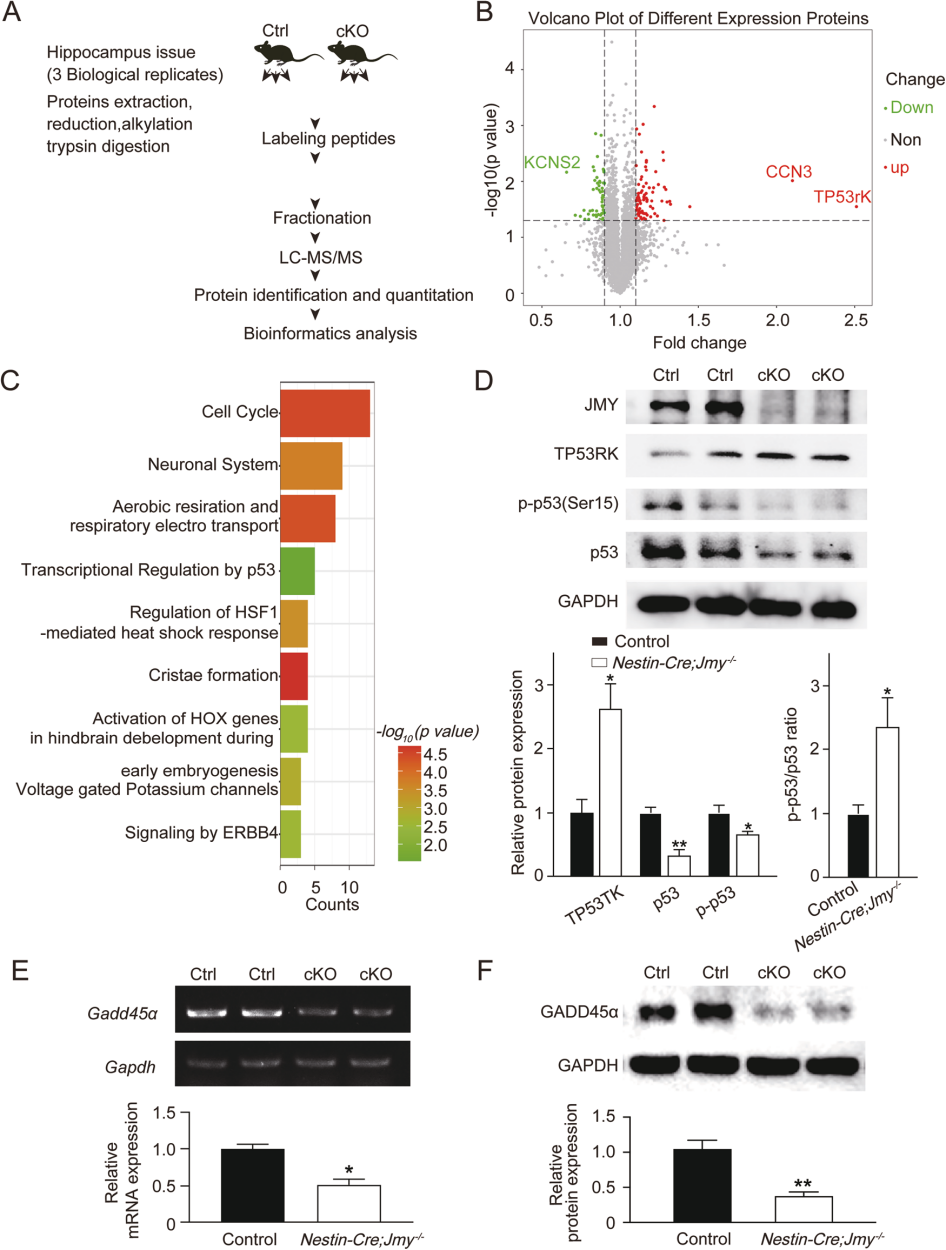
Change of p53 downstream target genes in *Nestin-Cre;Jmy*^*−/−*^ mouse brain. **A** Workflow diagram. Proteins from brain tissue were subjected to TMT labeling followed by mass spectrometric analysis. Ctrl, control; cKO, conditional knockout; **B** Volcano plot showing the distribution of proteins identified from *Nestin-Cre;Jmy*^*−/−*^ and control mice quantified by proteomic analysis. Proteins with significant differential expression are highlighted. Black, no significant change; Green, downregulated expression; Red, upregulated expression. **C** Functional enrichment analysis of differentially expressed proteins was performed with Metascape. The −log10 adjusted *p* value reflects the statistical significance of enrichment. **D** Western blot analysis of TP53RK, p53, and phosphorylated p53 at Ser15 (p-p53) expression in control (Ctrl) and conditional knockout (cKO, *Nestin-Cre;Jmy*^*−/−*^) mice. GAPDH was used as an internal control. *n* = 4 for each group, * *p* < 0.05, ** *p* < 0.01, mean ± SEM. **E** RT–PCR and quantitative real-time PCR analyses of *Gadd45α* mRNA levels in brain lysates from control and *Nestin-Cre;Jmy*^*−/−*^ mice (*n* = 4 for each group, * *p* < 0.05, mean ± SEM). GAPDH was used as an internal control. **F** Western blot analysis of GADD45α expression in control and *Nestin-Cre;Jmy*^*−/−*^ mice. GAPDH was used as an internal control. (*n* = 4 for each group, ** *p* < 0.01, mean ± SEM).

To explore the molecular mechanisms underlying the observed defects and in view of the potential function of JMY in learning and memory, we performed quantitative proteomics analysis of the control and *Nestin-Cre;Jmy*^*−/−*^ mice. We identified 150 differentially expressed proteins (87 upregulated while 63 downregulated; Fig. 8A, B). Notably, TP53RK was the most upregulated protein in the cKO mice, which is known to regulate p53 phosphorylation [34] (Fig. 8B). These differentially expressed proteins were significantly enriched in processes such as cell cycle, neuronal system and transcriptional regulation by p53. Among these pathways, the cell cycle pathway involved the largest protein subset (Fig. 8C). As JMY is a coactivator of p53, we focused on the p53-related pathways and examined the expression of key p53 downstream target genes involved in cell cycle regulation. Western blot analysis revealed that TP53RK expression and the ratio of p53 phosphorylation (Ser15) to total p53 were significantly increased, whereas the total p53 protein level was markedly reduced in *Nestin-Cre;Jmy*^*−/−*^ mice compared to control littermates (Fig. 8D). This dual phenotype suggests a complex regulation, in which JMY deficiency destabilizes basal p53 protein while simultaneously enhancing phosphorylation-mediated activation [10]. We further investigated mRNA level changes in representative p53 downstream targets involved in cell cycle regulation in the brain of *Nestin-Cre;Jmy*^*−/−*^ mice, specifically *p21*(a G1/S checkpoint regulator) [35] and *Gadd45α* (a G2/M checkpoint regulator) [36]. Validation experiments revealed that while *p21* expression showed no significant change (Fig. S2), *Gadd45α* was significantly downregulated (Fig. 8E, F). In addition to these primary analyses, we also examined several other p53 downstream effectors (*Btg2*, *Fbxw7*, *Sfn*, *Puma*, *Bcl2*, *Bax*), with results provided in the Supplementary Information (Fig. S2). These results indicate that JMY deficiency disrupts cell cycle dynamics via p53 signaling and reduced *Gadd45α* expression, potentially contributing to abnormal neuronal processes affecting learning and memory.

## Discussion

During cortical development, neural progenitors in the ventricular and subventricular zones (VZ/SVZ) generate diverse populations of projection neurons through tightly regulated proliferation, migration, and differentiation [2, 37]. In this study, we found that JMY is abundantly expressed in the brain, begins to be expressed in the cortex in the early embryo, is distributed mainly in the VZ/SVZ, and shows transient high expression during the later stages of embryo development (Fig. 1). The specific spatiotemporal expression pattern of JMY suggests that it is important for the regulation of neuronal development. Moreover, JMY, as a co-factor of p53, is abundantly expressed in the nucleus of neurons (Fig. 2). Previous studies have revealed that p53-deficient mouse embryos exhibit severe malformations during cortical development, such as cortical stratification disorders, local hyperplasia, and exencephaly [38]. p53 can inhibit the proliferation and renewal capacity of neural stem cells by regulating the cell cycle [12], and it regulates the neuronal differentiation kinetics and maturity of neurons [14].

These findings suggest that JMY plays an important role in the proliferation and differentiation of neurons. To verify this, we used BrdU-Ki67 cell cycle profiling assay and showed that knockdown of JMY promoted proliferation and suppressed terminal mitosis and differentiation of neural progenitor cells, suggesting that JMY can cause progenitor cells to shift from proliferative cell division to differentiative cell division. This finding was further supported by an increase in the abundance of proliferating neural progenitor cells (Nestin-positive cells) with the knockdown of JMY. The size of the neural progenitor cell pool is regulated by proliferative divisions that expand the pool and differentiative divisions that reduce the pool [39, 40]. These findings indicate that the expense of the neural progenitor cells in the pool is reduced.

In the developing mammalian brain, neural progenitor cells in the VZ/SVZ have the ability to actively proliferate and differentiate, and their cell cycle status affects subsequent neuronal migration and localization to different layers of the cerebral cortex [41]. If G2/M phase arrest fails to effectively promote cell cycle termination, progenitor cells may remain in a proliferative state, making it difficult to transition into migrating neurons, thereby affecting normal neuronal migration and the establishment of cortical laminar structures [42]. Moreover, as an actin nucleation factor [9], JMY has the potential to regulate cell dynamics and affect cell morphogenesis. In the present study, we used RNAi to knock down the expression of *Jmy* in the embryonic stage and found that the migration of neurons in the lower layer was significantly impaired, whereas the overexpression of JMY promoted the migration of cerebral cortical neurons (Fig. 3). These findings suggest that JMY plays an important role in the migration of neurons in the developing brain by promoting differentiative cell divisions. Therefore, we speculate that JMY regulates neuronal migration by affecting the ability of p53 to regulate proliferation and differentiation, inhibiting the proliferative division of neural progenitor cells, promoting exit from the cell cycle, and activating the migration process.

Because reduced differentiation and delayed migration typically indicate impaired neuronal maturation, we next examined whether JMY deficiency also affects dendritic morphogenesis, a key hallmark of neuronal structural maturation. In utero electroporation experiments revealed that JMY-deficient neurons exhibited reduced dendritic length, fewer branches, and simplified arborization, indicating defects in neuronal structural maturation.

In adult cKO mice, loss of JMY further resulted in striking cortical lamination defects. Both *Nestin-Cre;Jmy*^*−/−*^ and *Emx1-Cre;Jmy*^*−/−*^ mice displayed disorganized distributions of CUX1^+^ neurons, demonstrating that JMY is critical not only during progenitor proliferation and migration but also for maintaining laminar organization of the mature cortex.

Disruptions in these processes can lead to cognitive impairments and neurodevelopmental disorders [1, 2]. Functional consequences were evident behaviorally, as spatial learning and memory were impaired in both *Nestin-Cre* and *Emx1-Cre* lines (Morris water maze, Y-maze), thereby linking JMY-dependent developmental programs to cortical cognitive function. We suggest that the impaired learning and memory of the JMY-deficient mice could be attributed to delayed neuronal differentiation and migration, which may reduce the number of fully developed and functional neurons and disrupt the establishment of neural circuits. Other members of the WASP family have also been implicated in learning and memory functions [43, 44], further supporting the importance of WASP family proteins in neurodevelopment and cognitive functions. Therefore, the spatial learning and memory deficits caused by JMY deletion are reminiscent of findings from other WASP family members, underscoring the essential role of this protein family in maintaining normal cognitive functions. Further investigation of JMY may provide new insights into the molecular basis of neurodevelopmental disorders and potential therapeutic strategies.

Notably, our proteomic and validation analyses revealed that both TP53RK expression and the proportion of phosphorylated p53 were significantly increased in *Jmy-*cKO mice, suggesting enhanced p53 transcriptional activation. However, we simultaneously observed a pronounced reduction in basal p53 protein levels, which could potentially compromise its transcriptional activity. To further explore the integrated effects of JMY loss, we examined the expression of key downstream p53 targets, specifically *p21* and *Gadd45α*. We found that *p21* expression remained unchanged, whereas both the mRNA and protein levels of GADD45α were significantly reduced. Given that GADD45α promotes G2/M-phase cell cycle arrest [45, 46], our findings indicate that the net effect of JMY deficiency is a loss of cell cycle inhibition and enhanced cell proliferation, consistent with our previous experimental results (Fig. 4). Loss of GADD45α-mediated G2/M arrest would allow progenitor cells to bypass essential quality-control steps and may undergo DNA replication without completing normal cell division, leading to the abnormal accumulation of Nestin-positive progenitor cells in the proliferative zone [47–49]. These cells are unable to exit the cycle smoothly and enter the differentiation pathway, resulting in an overall decline in functional proliferative efficiency [50]. Therefore, the observed increase in Nestin-positive cells in this study may reflect a state of undifferentiated and continuous proliferation, consistent with the observed decrease in the normalized cell cycle exit index and the increase in BrdU incorporation ratio [51]. This paradoxical phenotype suggests the presence of complex signal network interactions in the JMY-deficient model. Some studies have previously reported that JMY knockdown promotes p53 degradation [11, 20], while others showed that JMY depletion does not alter p53 protein abundance but instead impairs its transcriptional activity in certain cell lines [52]. Taken together with our data, these findings suggest that the role of JMY in p53 regulation may be context-dependent, influenced by cell- or tissue-specific factors. Moreover, our results raise the possibility that reduced basal p53 levels may trigger compensatory enhancement of p53 phosphorylation and transcriptional activation. Thus, the regulatory effects of JMY on p53 function likely vary across cellular environments and require context-specific experimental validation. Additionally, experimental validation is needed to determine whether other p53 downstream targets are also dysregulated at the post-transcriptional or post-translational level, thereby refining the causal chain in this regulatory network and advancing our understanding of the mechanisms underlying neurodevelopmental abnormalities.

In this study, we show that JMY contributes to several aspects of cortical development. Loss of JMY delayed neuronal migration, impaired cell cycle exit, and disrupted neuronal differentiation, ultimately leading to deficits in spatial learning and memory. These findings underscore the regulatory role of JMY in neural development, deepen our understanding of cortical development, and provid novel insights into the p53-dependent mechanisms and potential relevance to neurodevelopmental disorders.

## Materials and methods

### Animals

C57BL/6 mice were obtained from the animal breeding colony of the Animal Centre of Guangxi Medical University, China. Due to the embryonic lethality associated with global knockout of *Jmy*, we utilized *LoxP-*flanked *Jmy* mice, which were generated by inserting *LoxP* sequences flanking exon 3 of the *Jmy* gene via homologous recombination. This approach resulted in the creation of *Jmy*^*loxP/loxP*^ mice, which were then used to generate *Jmy* conditional knockout (cKO) mice using Cre/*loxP* technology. The construction of *Jmy*^*loxP/loxP*^ mice was performed by Shanghai Biomodel Organism Science and Technology Development Co., Ltd. (Animal Experimentation License No. SCXK (HU) 2017-0010).

These *Jmy*^*loxP/loxP*^ mice were then crossed with *Nestin-Cre* and *Emx1-Cre* transgenic mice. *Nestin-Cre* mice, obtained from Shanghai Biomodel Organism Science and Technology Development Co., Ltd., express Cre recombinase under the control of the *Nestin* promoter, which is predominantly active in neural progenitor cells throughout the developing central nervous system [53]. By crossing *Jmy*^*loxP/loxP*^ mice with *Nestin-Cre* mice, *Jmy* was specifically deleted in neural progenitor cells, generating *Nestin-Cre;Jmy*^*−/−*^ mice. In contrast, *Emx1-Cre* mice, which were sourced from The Jackson Laboratory (Stock No: 005628), express *Cre* recombinase under the control of the *Emx1* promoter, which is expressed in early forebrain progenitors and has strong activity in the cortex and hippocampus [54]. Crossing *Jmy*^*loxP/loxP*^ mice with *Emx1-Cre* mice resulted in the conditional deletion of *Jmy* in the cortex and hippocampus, generating *Emx1-Cre;Jmy*^*−/−*^ mice. All the mice were housed in temperature-controlled rooms under a 12 h light/12 h dark cycle and were given food and water *ad libitum*. All the studies were approved by the Institutional Animal Care and Use Committee of Guangxi Medical University (Approval No. 201809001) and were performed in compliance with the U.S. National Institutes of Health Guide for the Care and Use of Laboratory Animals.

### RNA extraction and real-time PCR

The brain tissues were homogenized with TRIzol Reagent (Invitrogen) at 4 °C. RNA was extracted according to the manufacturer’s instructions. The final RNA pellet was suspended in diethylpyrocarbonate (DEPC)-treated water, and 2 μg of total RNA was then subjected to reverse transcription using oligo (dT) primers and Moloney murine leukemia virus (M-MLV) transcriptase (Invitrogen). Real-time PCR was performed with a LightCycler 480 Real-Time PCR System (Roche) according to the manufacturer’s instructions. Starting RNA levels were quantified by using *Gapdh* as the external standard. The primer sets were chosen from PrimerBank, and the gene sequences are available in the GenBank database. The primers used for gene expression analysis are listed in Supplementary Materials. The primers used were synthesized by Sunny Biotech.

### Western blot analysis

Tissues were homogenized as previously described [55]. All protein samples were separated via 10% SDS‒polyacrylamide gel electrophoresis (Bio-Rad) and blotted onto PVDF membranes (Millipore). The membranes were blocked with 5% nonfat milk in 0.05% Tween 20 at room temperature for 1 h and probed with anti-JMY (Santa Cruz Biotechnology), anti-TP53RK (Abcam), anti-Phospho-p53 (Cell Signaling Technology), anti-p53 (Cell Signaling Technology), anti-GADD45α (Santa Cruz Biotechnology) and HRP-coupled mouse anti-GAPDH (Aksomics) antibodies. The secondary antibody used for JMY was anti-goat IgG coupled to HRP (Aksomics). The bands were visualized by enhanced chemiluminescence (TIANGEN). Band intensities were measured with ImageJ software.

### Immunohistochemistry

Forebrain sections from mice at different developmental stages were postfixed in 4% paraformaldehyde (PFA). After washing, sections were incubated in blocking buffer (5% (wt/vol) bovine serum albumin (BSA), 10% (vol/vol) normal goat serum (NGS), 0.25% (vol/vol) Triton X-100) for 1 h to block nonspecific background staining. The sections were further incubated with primary antibodies against JMY (1:500, Santa Cruz), MAP2 (1:2000, Sigma), GFP (1:2,000, Invitrogen), GFAP (1:1000, Millipore), NeuN (1:1000, Millipore), CTIP2 (1:200, Abcam), CUX1 (1:200, proteintech) and Nestin (1:500, Sigma) overnight at 4 °C. After being washed, the sections were incubated with their respective secondary antibodies at room temperature for 2 h and then counterstained with Hoechst 33342 for 10 min at room temperature before being covered with coverslips.

Cultured neurons were fixed with 4% PFA and then immunostained with primary antibodies against JMY (1:500, Santa Cruz), MAP2 (1:2000, Sigma), GFP (1:2000, Invitrogen), GFAP (1:1000, Millipore), and NeuN (1:1000, Millipore) overnight at 4 °C. After washing, the cells were incubated with their respective secondary antibodies at room temperature for 2 h and then counterstained with Hoechst 33342 and phalloidin for 10 min at room temperature.

For DAB staining, brain sections (30 μm) were incubated in 0.01 M phosphate-buffered saline (PBS) supplemented with 3% hydrogen peroxide for 10 min to block endogenous peroxidase activity and then in blocking buffer containing 5% BSA/10% normal goat serum/0.25% Triton X-100 for 60 min at room temperature to prevent nonspecific staining. IHC was subsequently conducted on these free-floating sections, and staining was visualized with a standard ABC Elite kit (Vector Labs).

### In situ hybridization

In situ hybridization of the brain sections was performed with digoxigenin-labeled antisense riboprobes. The full-length cDNA of *Jmy* was amplified with specific PCR primers and subsequently cloned and inserted into the pGEM-T easy vector (Promega) to generate an antisense probe for *Jmy*. The digoxigenin-labeled antisense riboprobes were synthesized by in vitro transcription via the SP6 Riboprobe System (Promega).

The mice were perfused with 4% paraformaldehyde (PFA) and post-fixed in 4% PFA at 4 °C. Fixed brains were cryoprotected overnight in 15–30% sucrose/PBS at 4 °C, mounted in optimal cutting temperature (OCT) compound and sectioned coronally (20 μm) with a cryostat (Leica). The brain sections were hybridized for 18 h at 60 °C. The hybridization signal was detected with anti-DIG–alkaline phosphatase Fab fragments (Roche) and nitro blue tetrazolium chloride (NBT) plus 5-bromo-4-chlor-indolyl-phosphate (BCIP) as substrates for the color reaction.

### In utero electroporation

In utero electroporation was performed as described previously [24], with minor modifications. Briefly, pregnant mice at E13.5 or E14.5 were anesthetized with pentobarbital sodium. Midline laparotomy was performed after the abdomen was cleaned, and the uterus was exposed. DNA plasmids (1 μL) at a high concentration were injected into the lateral ventricle with 0.05% Fast Green (Sigma) through a polished micropipette. Square electric pulses were delivered to the embryos through the uterus at a rate of one pulse per second by holding the embryos with forceps-type electrodes while the uterus was kept wet by dropping saline (prewarmed at 37 °C) between the electrodes. Five electrical pulses (50 V, 50 ms, 1 s interval) were applied across the uterine wall using an ECM-830 BTX square wave electroporator. The uterine horns were then returned to the abdominal cavity, and the abdomen wall and skin were sutured using a surgical needle and thread.

### BrdU labeling

For bromodeoxyuridine (BrdU) incorporation, E15.5 mice were intraperitoneally injected with 100 mg/kg BrdU (Sigma) and sacrificed at birth. The cryostat sections of the brains were incubated in 1 N HCl for 30 min at 37 °C to denature the DNA and then neutralized in 0.1 M borate buffer (pH 8.0). The sections were then incubated with rat anti-BrdU (1:100, Sigma) and rabbit anti-Ki67 (1:400, Invitrogen) antibodies after being washed with 0.01 M PBS (pH 7.4). A slice from the same plane was selected as the observation object for each mouse, and the numbers of BrdU-positive cells and BrdU and GFP double-positive cells were counted.

### Nissl staining

For assessment of gross brain morphology, coronal and sagittal sections (30 µm) were prepared from control and conditional knockout mice. Nissl staining was conducted on brain sections as described previously [56]. Images were captured under a Leica DMi8 inverted microscope using Neurolucida software (MBF Bioscience).

### Dendritic reconstruction

Neurons were imaged under bright-field optics and traced in Neurolucida (MBF Bioscience). Quantification was performed in Neurolucida software to obtain total dendritic length, number of branch points, and terminal tips.

### Behavior tests

#### Morris water maze test

The standard procedure of the Morris water maze test was used [57]. Adult male mice (10–12 weeks old) of each genotype were trained to find the visible platform with four trials a day for the first day and were tested to find the hidden platform for 5 to 6 consecutive days. In each trial, the mice were allowed to swim until they found the hidden platform when they started from different, random locations around the perimeter of the tank. The mice were then allowed to sit on the platform for 10 s before being picked up. On the probe trial day, the platform was removed from the tank. The escape latency and the time spent in each quadrant were recorded by a video camera. The experimenter was blinded to the genotypes of the mice.

#### Y maze

The Y-maze design was based on published protocols, with modifications to adapt the system to mice [58, 59]. Briefly, the mice were placed into one of the arms of the maze (start arm) and allowed to explore the maze with one of the arms closed for 15 min (training trial). After a 1-h intertrial interval, the mice were returned to the Y maze by placing them in the start arm. The mice were subsequently allowed to explore all three arms of the maze freely for 5 min (test trial). The number of entries into and the time spent in each arm and the first choice of entry were determined from video recordings by an observer blinded to the genotype of the mice.

### Proteomic analysis

Proteins from hippocampi of the littermate controls and *Nestin-Cre;Jmy*^*−/−*^ mice (*n* = 3 per group) were extracted with SDT buffer, quantified, digested by trypsin, and labeled using TMT reagents. Peptides were fractionated by SCX chromatography, desalted, and analyzed by LC-MS/MS (Q Exactive coupled with Easy nLC). Raw MS data were identified and quantified with Mascot in Proteome Discoverer software against the UniProt mouse database, using the false discovery rate (FDR) < 1%. Differentially expressed proteins were defined by fold-change ≥1.10 and *p* < 0.05. Proteomic analysis was performed by Shanghai Applied Protein Technology Co., Ltd. (Shanghai, China). To gain more mechanistic insights, we restricted functional enrichment to reactome gene sets using Metascape. Finally, the results of enrichment analysis were visualized and displayed in graphical form to further understand the biological significance of each protein.

### Quantification and statistical analysis

For all statistical analyses, experimenters were blinded to genotypes of mice and the treatments of the animals or cells. Data are presented as mean ± SEM. Appropriate statistical methods were selected based on the data type. Student’s *t* test (two tailed), Fisher’s exact test, one-way ANOVA with Dunnett’s *post hoc* tests or Bonferroni’s tests were used for data analyses by SPSS 26.0 software. Statistical significance was defined as *p* < 0.05.

## Supplementary information

Supplementary

The primers used for real-time PCR

Full length western blots
