# Supplementary material for: Junction-mediating and regulatory protein (JMY) is a promoting protein for radial migration of cortical neurons
Source: Cell Death Discov. 2026 Feb 26;12:123. doi: 10.1038/s41420-026-02974-7 (PMC13031400; doi:10.1038/s41420-026-02974-7)
Supplement: Supplementary file 1 — Supplementary [file 41420_2026_2974_MOESM1_ESM.docx]

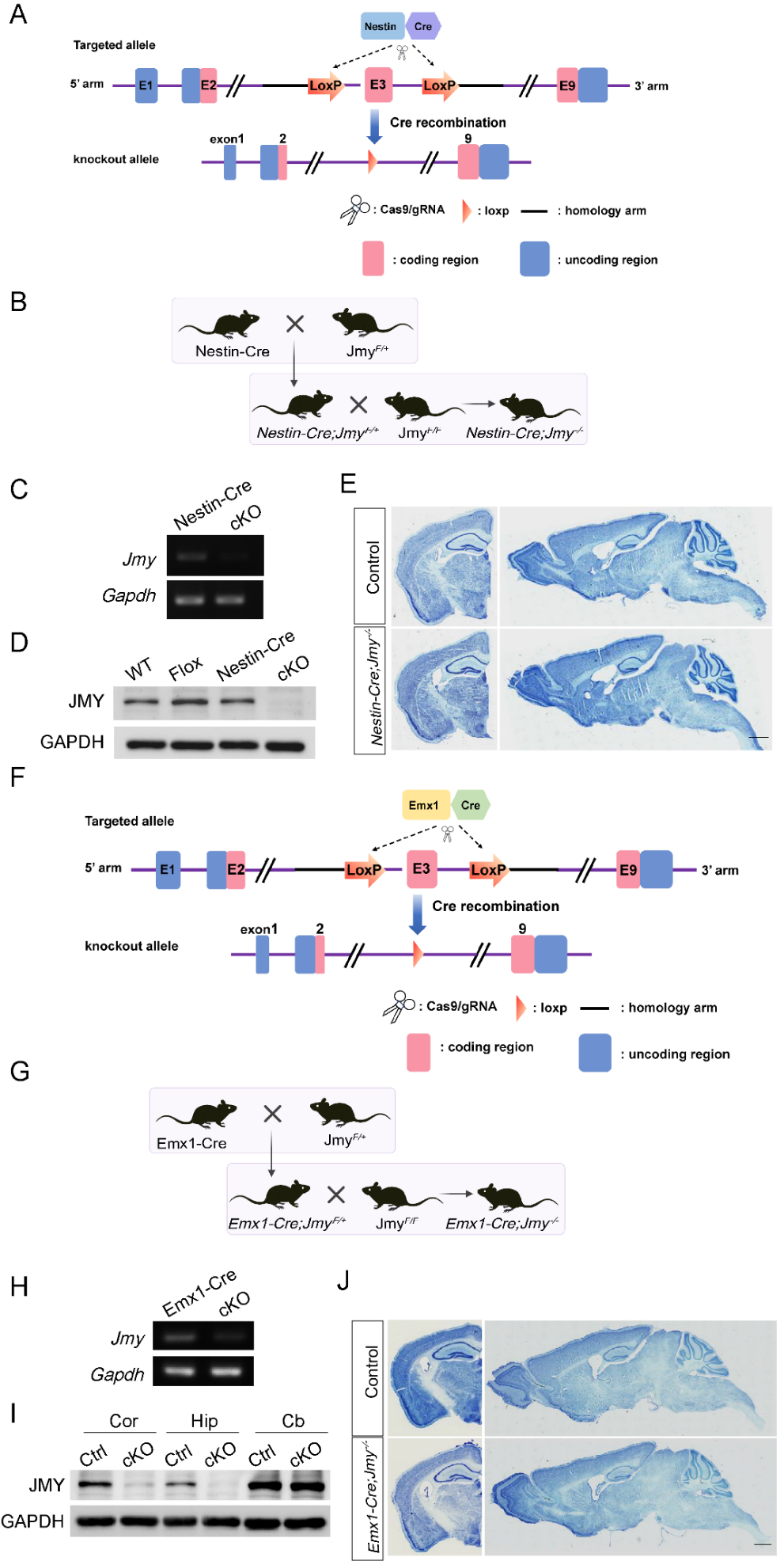


**Supplementary Fig. 1. Generation and validation of Jmy conditional knockout mice.**

**A** Schematic of the Jmy floxed allele and Cre-mediated recombination strategy used to generate *Nestin-Cre;Jmy^−/−^* mice. **B** Breeding strategy for *Nestin-Cre;Jmy^−/−^* mice. **C** RT–PCR analysis confirming loss of *Jmy* transcripts in *Nestin-Cre;Jmy^-/-^* cortex. Gapdh served as a loading control. **D** Western blot results showing the protein expression levels of JMY in the hippocampal tissues of *Nestin-Cre;Jmy^-/-^* (cKO), wild-type (WT), *Jmy^loxp/loxp^* (Flox), and *Nestin-Cre* mice. GAPDH was used as an internal loading control. **E** Representative Nissl staining of coronal and sagittal brain sections from control and *Nestin-Cre;Jmy^-/-^* mice showing no gross abnormalities in cortical or hippocampal morphology. Scale bars: 1 mm.
**F** Schematic of the Jmy floxed allele and Cre-mediated recombination strategy used to generate *Emx1-Cre;Jmy^-/-^* mice. **G** Breeding strategy for *Emx1-Cre;Jmy^-/-^* mice. **H** RT–PCR validation of *Jmy* deletion in *Emx1-Cre;Jmy^-/-^* mice cortex.**I** Western blot analysis of JMY protein expression in *Emx1-Cre;Jmy^-/-^* mouse brain. Ctrl,control; cKO, conditional knockout; Cor, cortex; Hip, hippocampus; Cb, cerebellum. GAPDH was used as an internal loading control. **J** Representative Nissl staining of coronal brain sections from control and *Emx1-Cre;Jmy^-/-^* mice, revealing normal gross brain morphology. Scale bars: 1 mm.


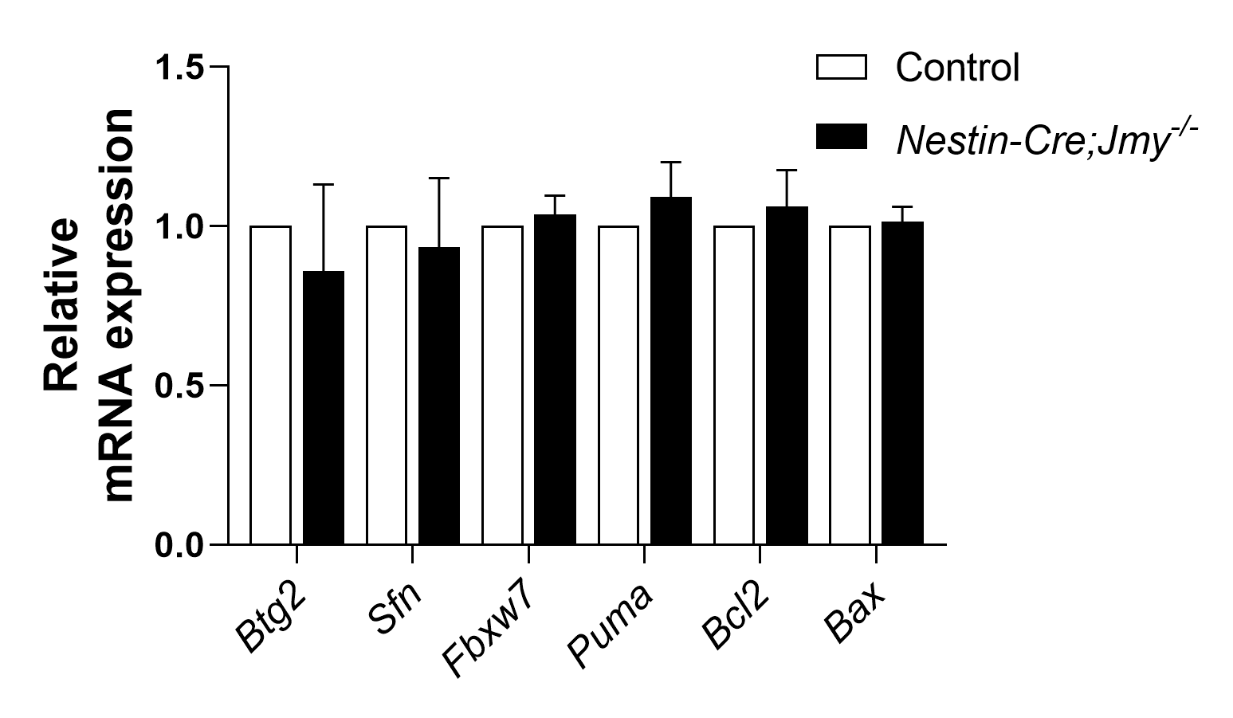


**Supplementary Fig. 2.** **Expression of p53 downstream target genes in control and *Nestin-Cre;Jmy^-/-^* mouse brains.**

Quantification of the mRNA levels of target genes downstream of p53 in brain lysates (*n* = 3 for each group, **p* < 0.05, mean ± SEM) via quantitative real-time PCR, with normalization to control group levels. GAPDH was used as an internal control.
