# Supplementary figures and images for: Junction-mediating and regulatory protein (JMY) is a promoting protein for radial migration of cortical neurons
Source: Cell Death Discov. 2026 Feb 26;12:123. doi: 10.1038/s41420-026-02974-7 (PMC13031400; doi:10.1038/s41420-026-02974-7)

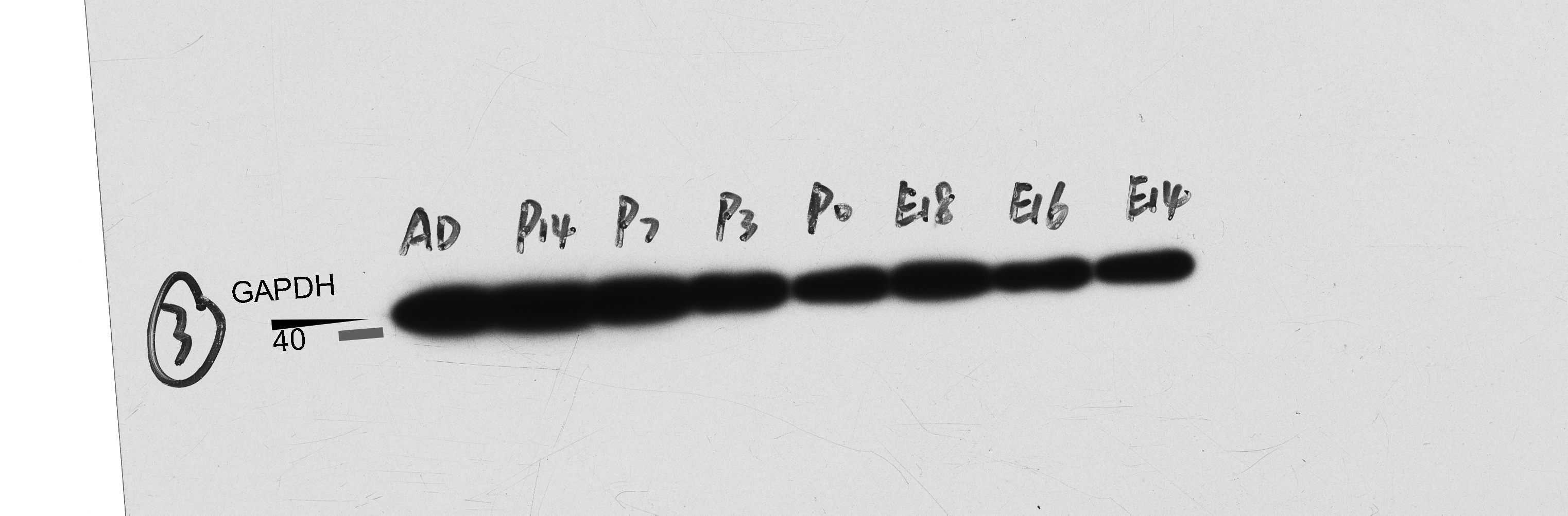

Supplement: Supplementary file 3 — Full length western blots [file 41420_2026_2974_MOESM3_ESM.zip › Fig.1E GAPDH reverse.tif]

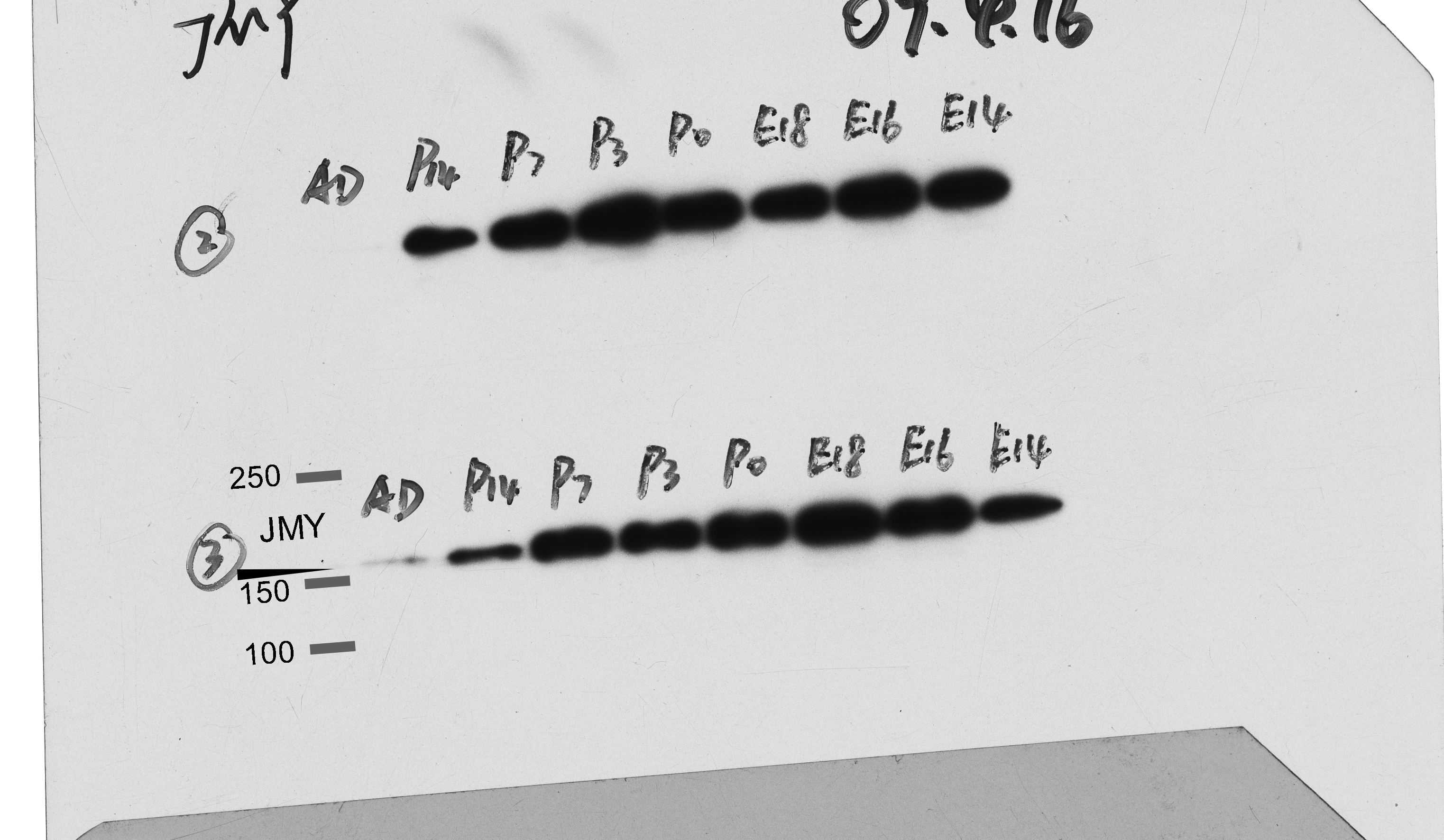

Supplement: Supplementary file 3 — Full length western blots [file 41420_2026_2974_MOESM3_ESM.zip › Fig.1E JMY reverse.tif]

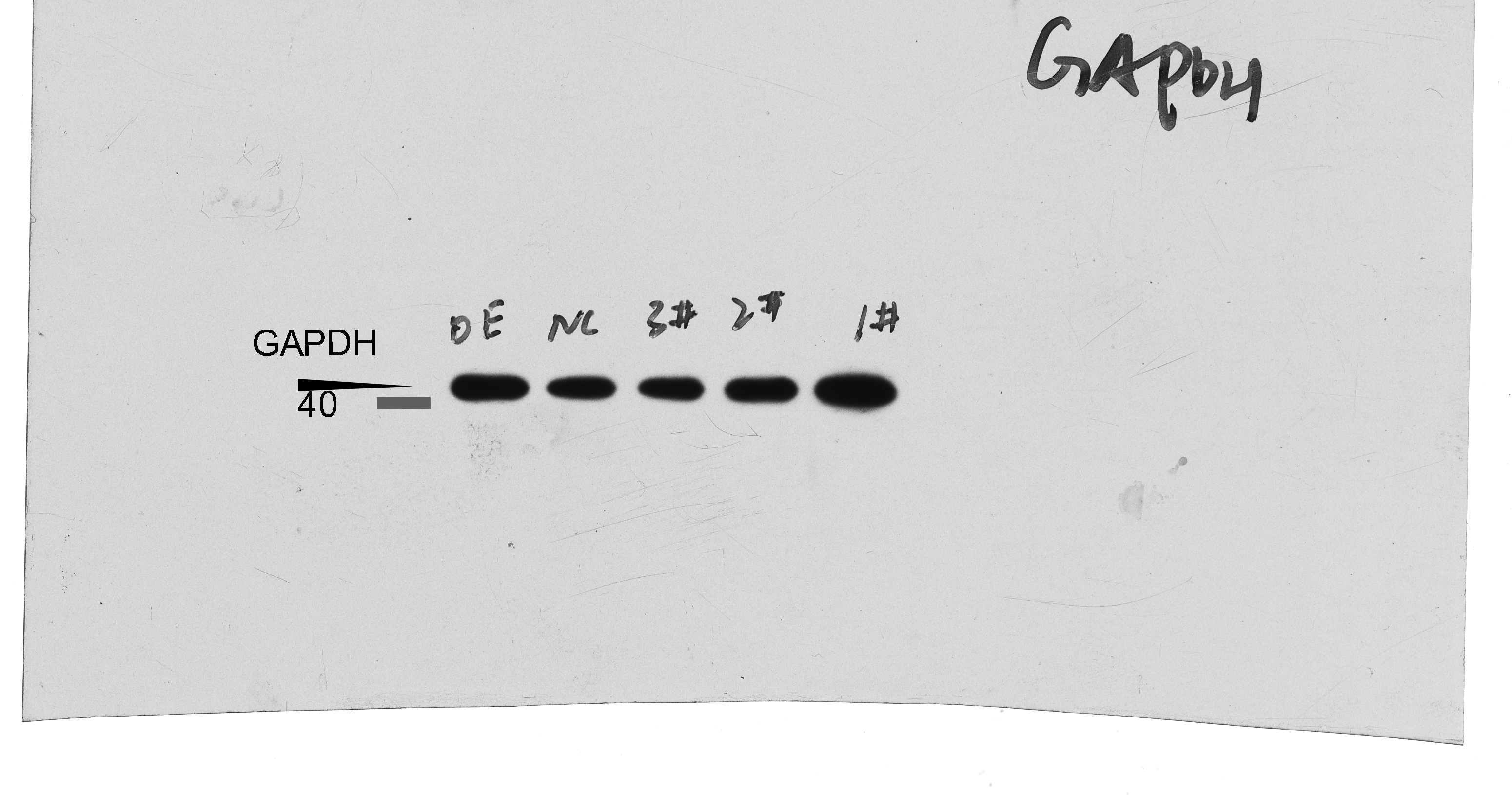

Supplement: Supplementary file 3 — Full length western blots [file 41420_2026_2974_MOESM3_ESM.zip › Fig.3A GAPDH-293T.tif]

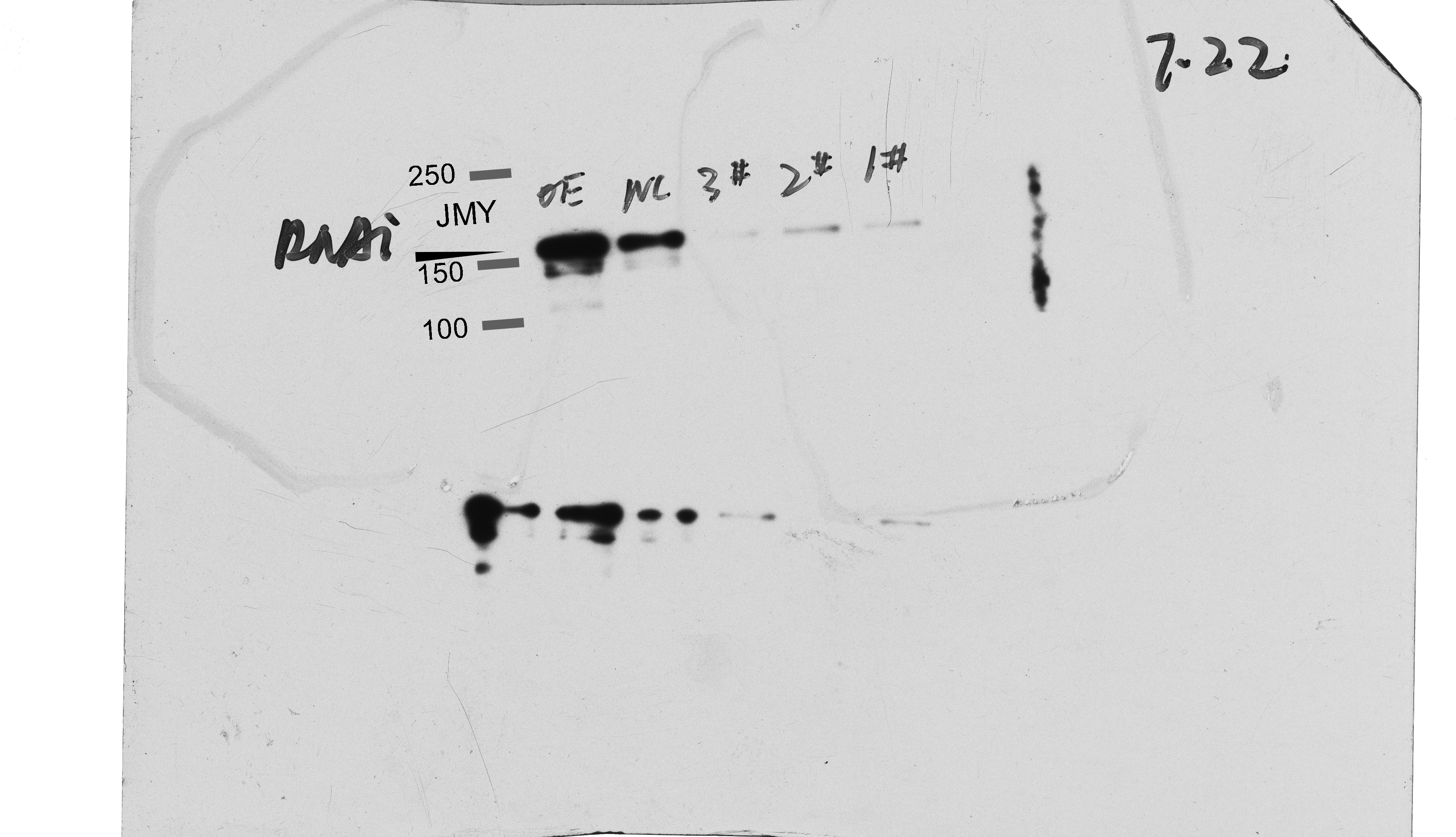

Supplement: Supplementary file 3 — Full length western blots [file 41420_2026_2974_MOESM3_ESM.zip › Fig.3A Jmy-siRNA-293T.tif]

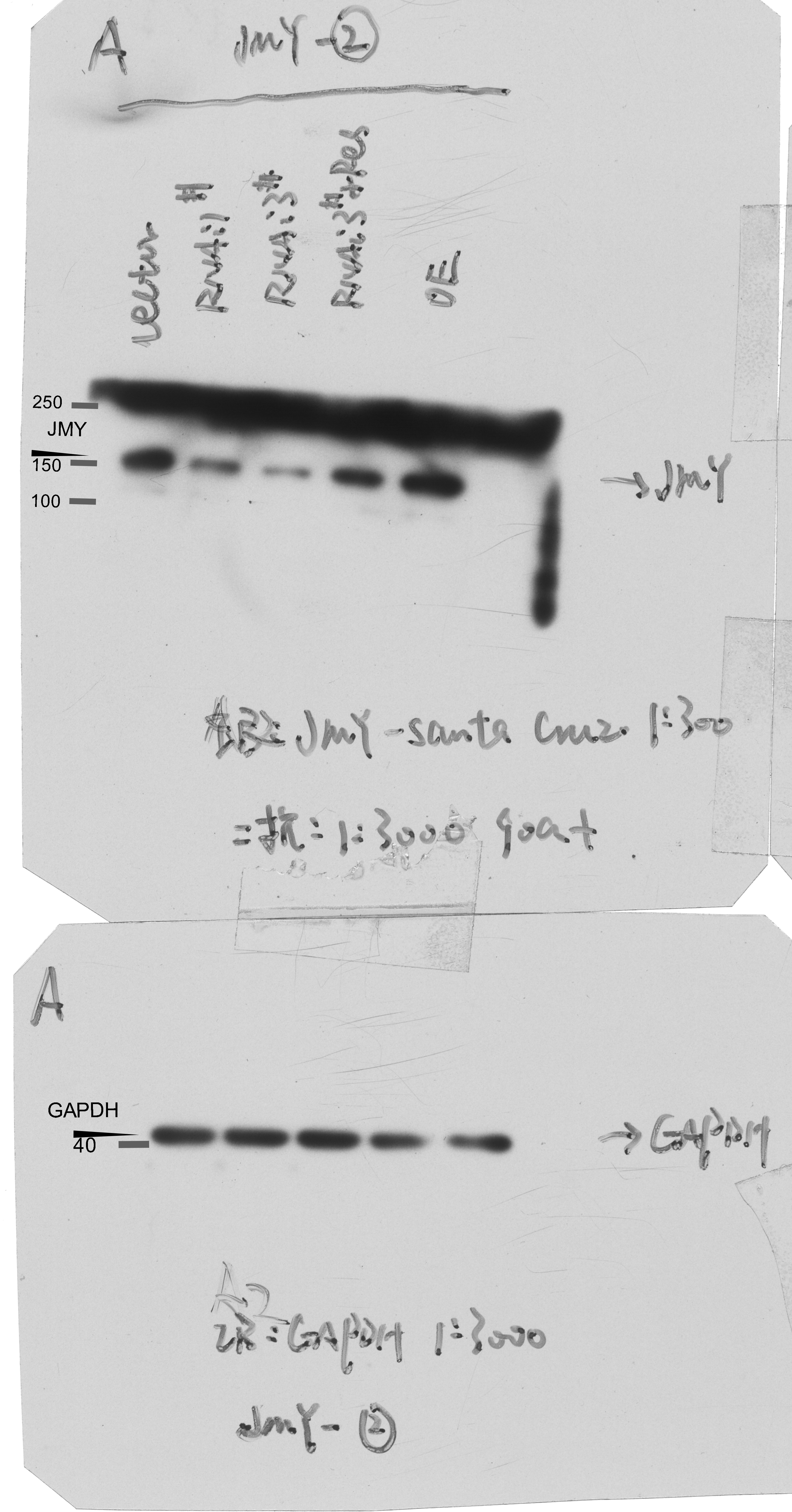

Supplement: Supplementary file 3 — Full length western blots [file 41420_2026_2974_MOESM3_ESM.zip › Fig.3A-JMY WB-RNAi OE RES1-DIV4 neuron.tif]

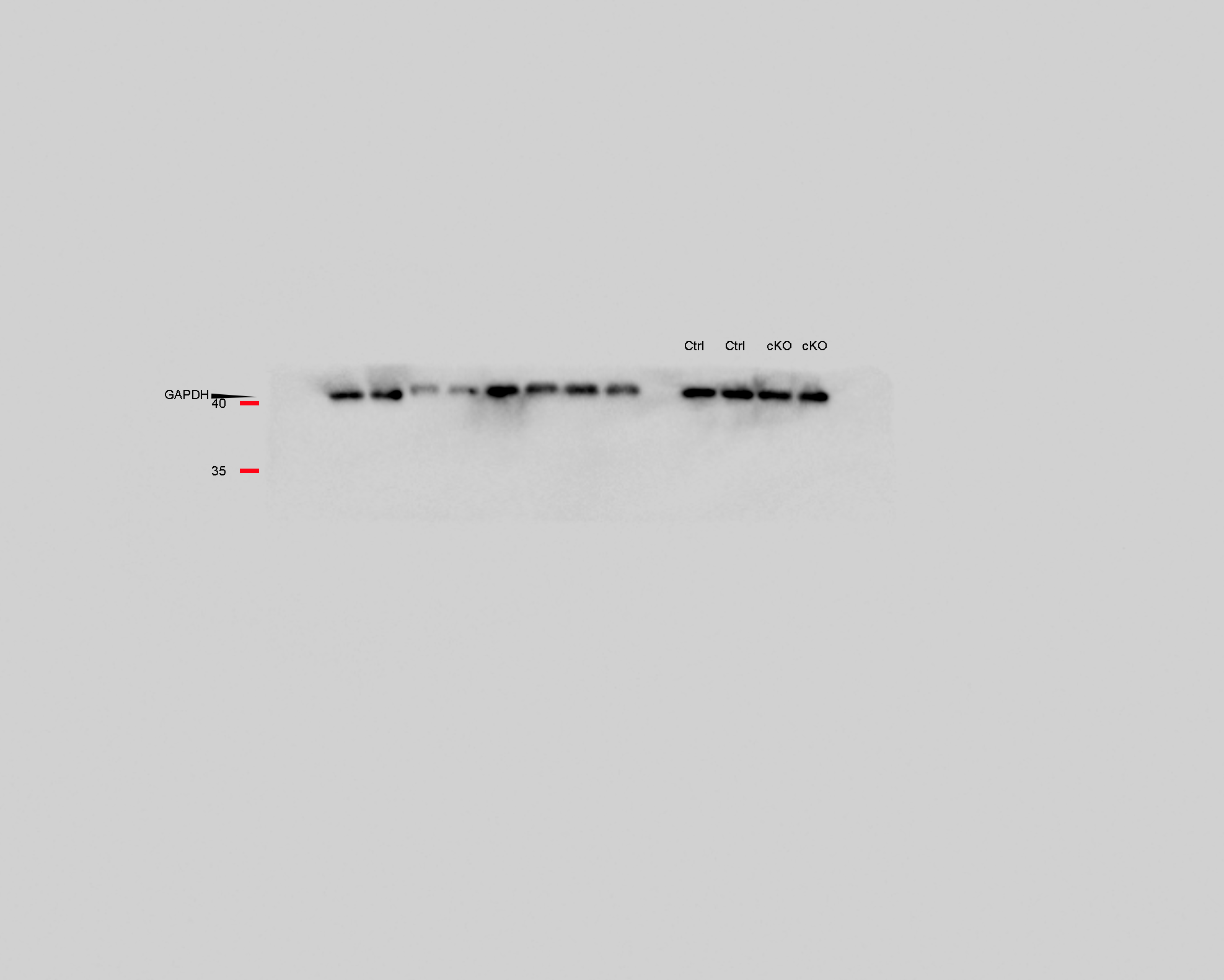

Supplement: Supplementary file 3 — Full length western blots [file 41420_2026_2974_MOESM3_ESM.zip › Fig.8D GAPDH.tif]

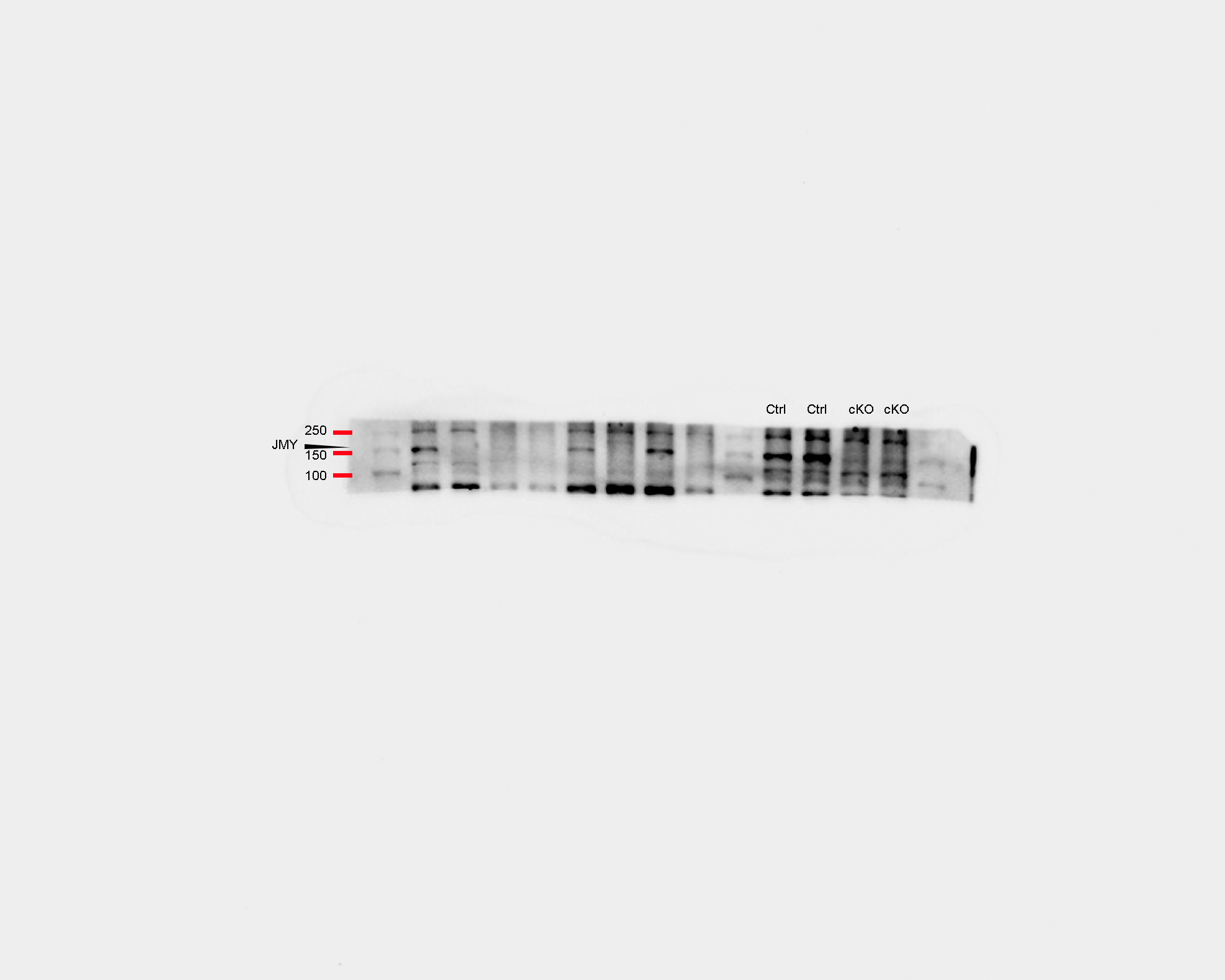

Supplement: Supplementary file 3 — Full length western blots [file 41420_2026_2974_MOESM3_ESM.zip › Fig.8D JMY.tif]

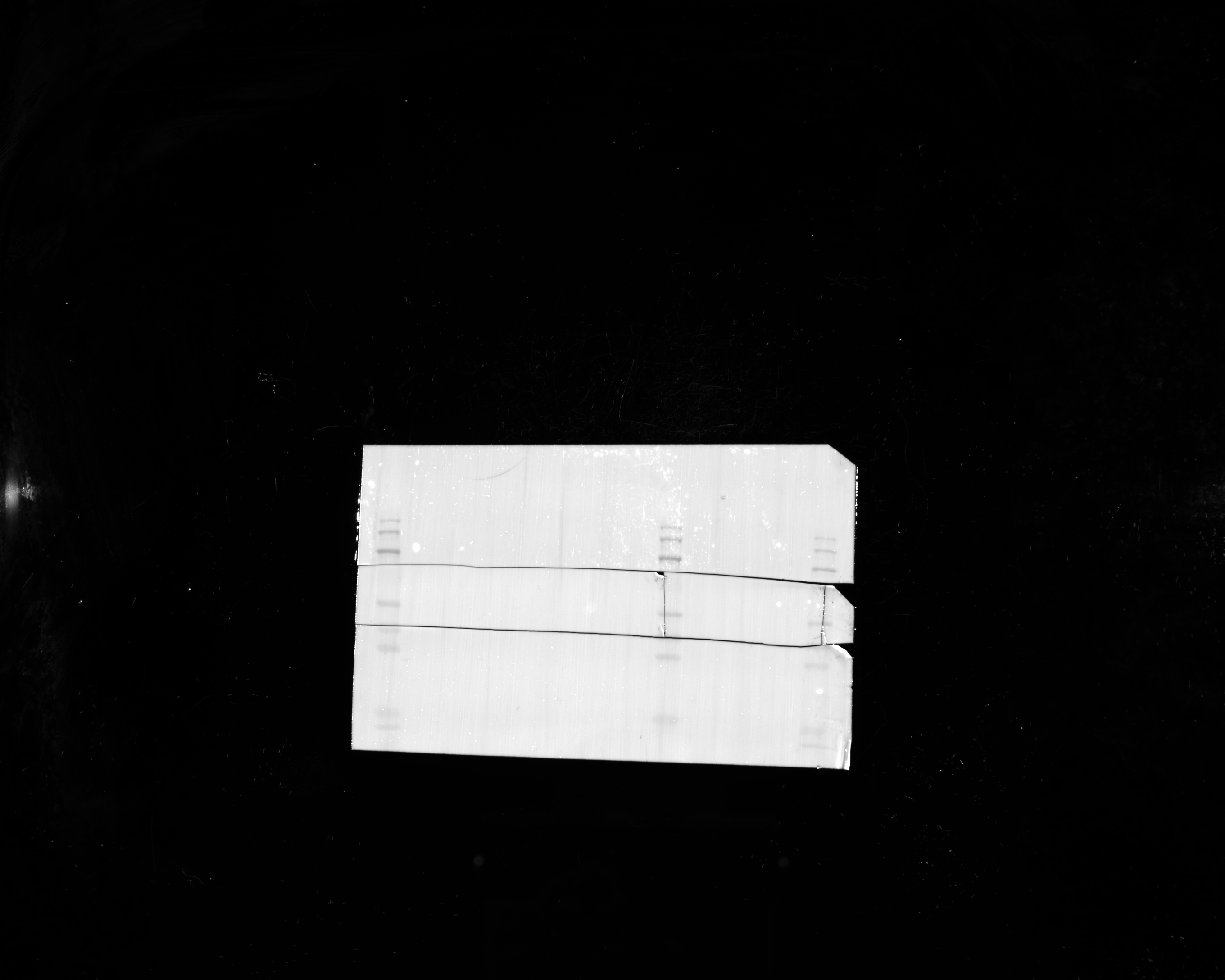

Supplement: Supplementary file 3 — Full length western blots [file 41420_2026_2974_MOESM3_ESM.zip › Fig.8D Splicing.tif]

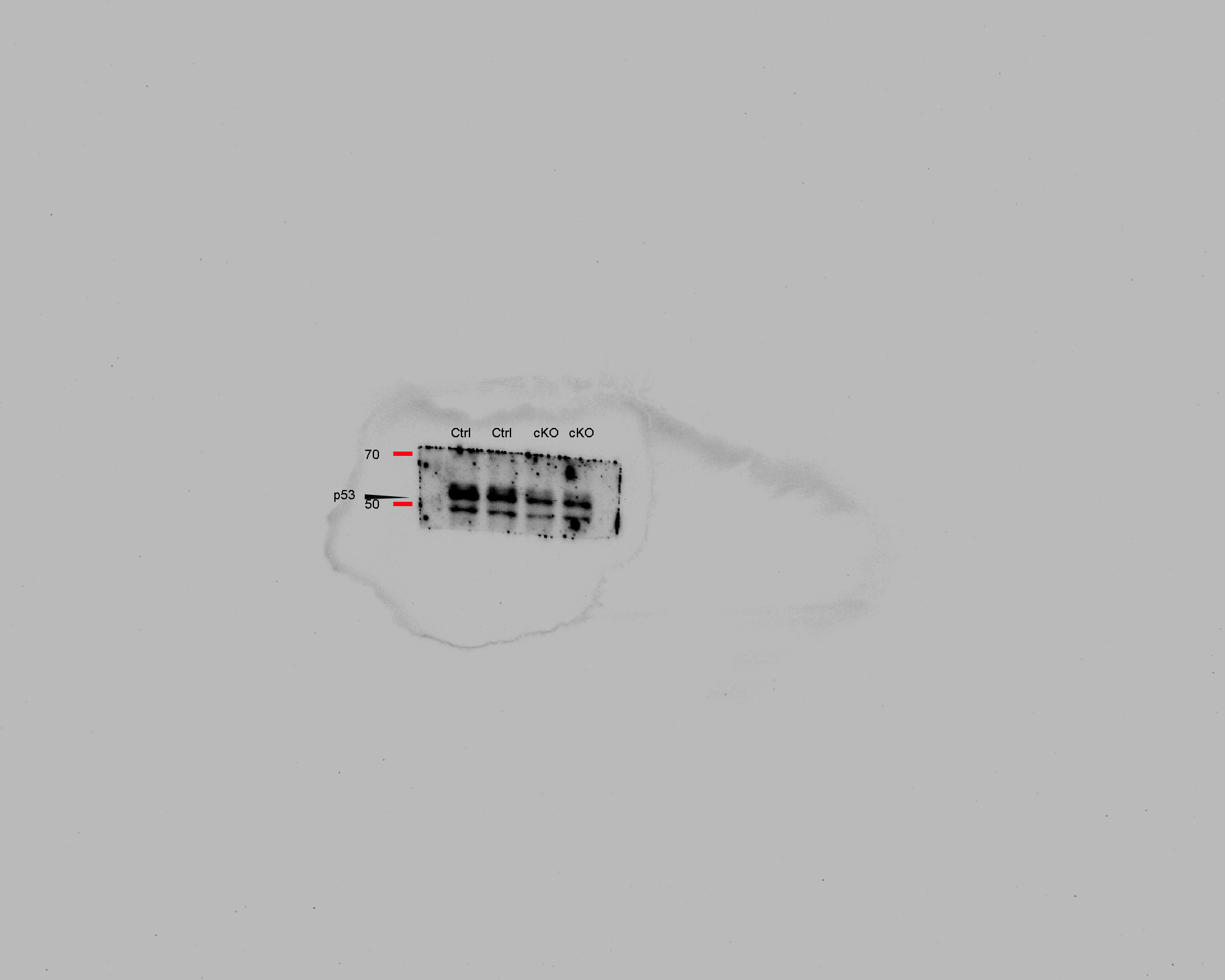

Supplement: Supplementary file 3 — Full length western blots [file 41420_2026_2974_MOESM3_ESM.zip › Fig.8D TP53.tif]

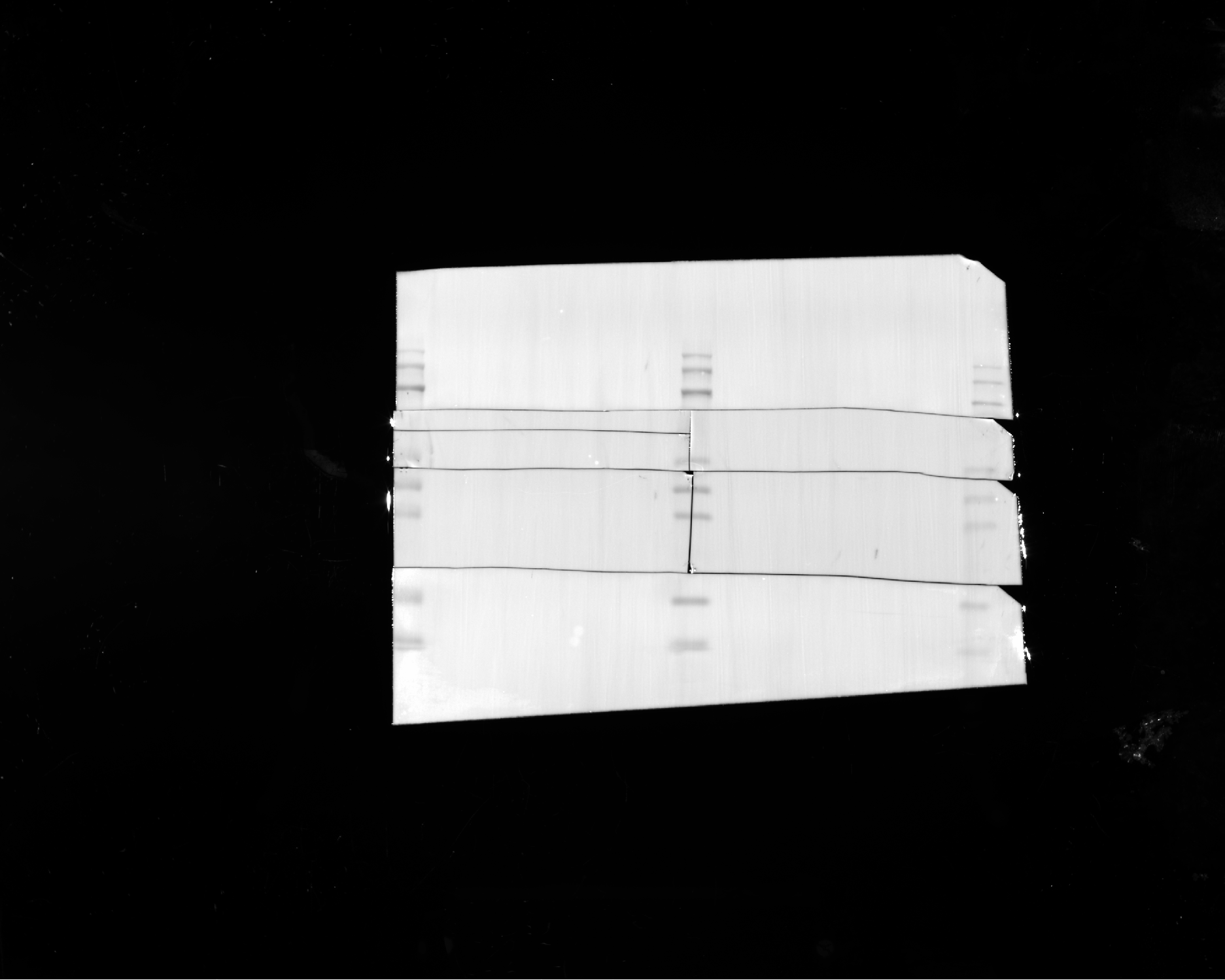

Supplement: Supplementary file 3 — Full length western blots [file 41420_2026_2974_MOESM3_ESM.zip › Fig.8D TP53RK splicing.tif]

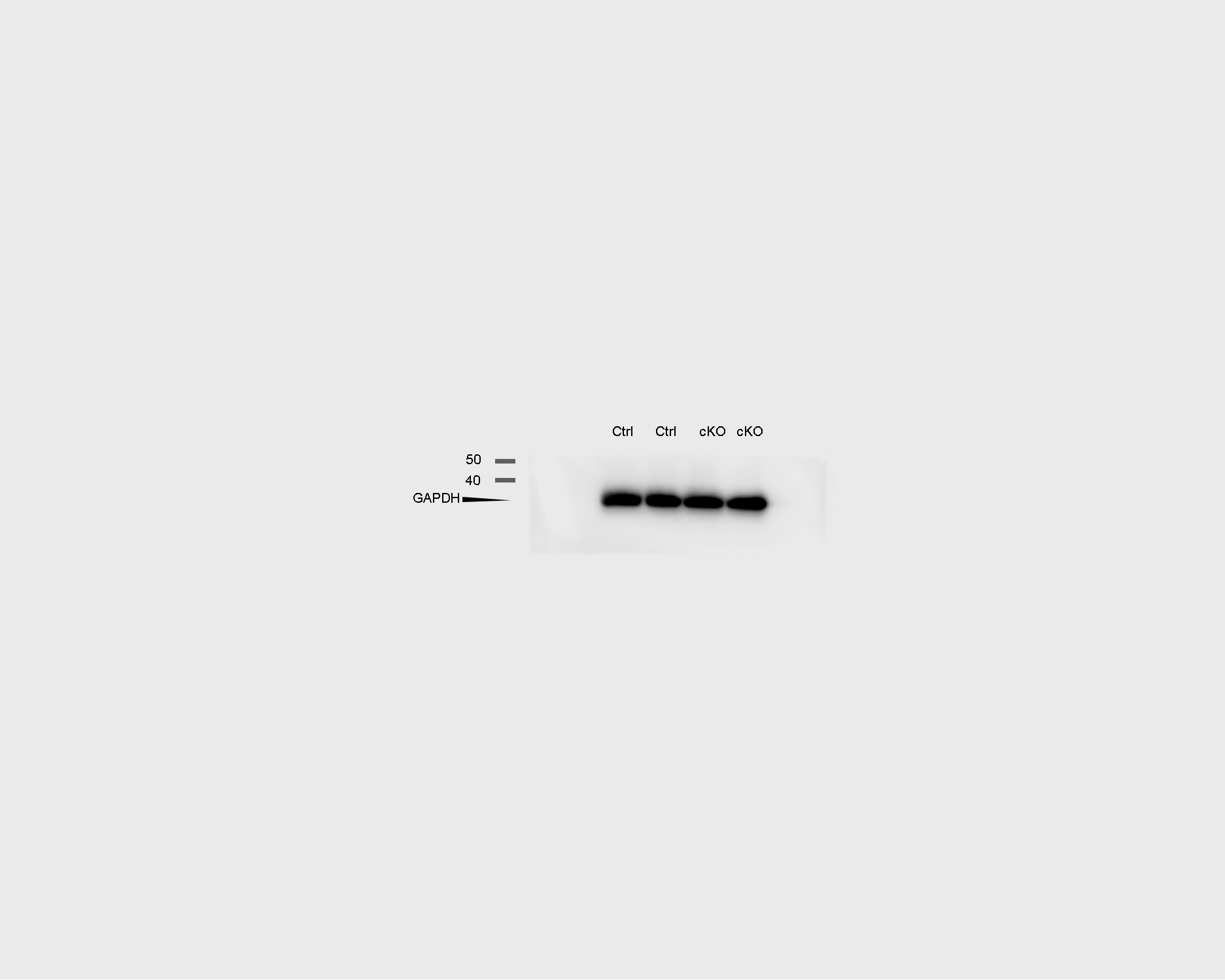

Supplement: Supplementary file 3 — Full length western blots [file 41420_2026_2974_MOESM3_ESM.zip › Fig.8D TP53RK-GAPDH.tif]

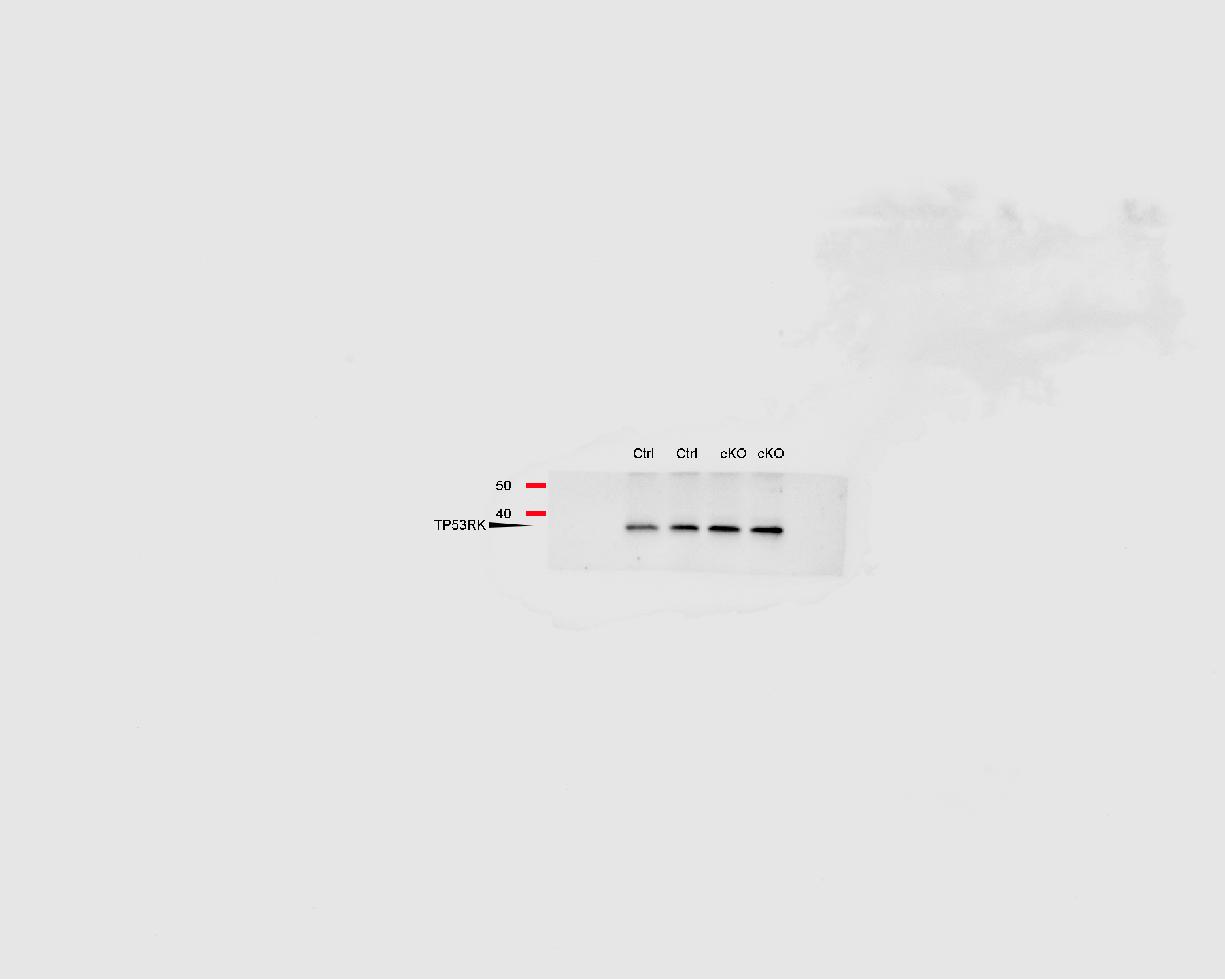

Supplement: Supplementary file 3 — Full length western blots [file 41420_2026_2974_MOESM3_ESM.zip › Fig.8D TP53RK.tif]

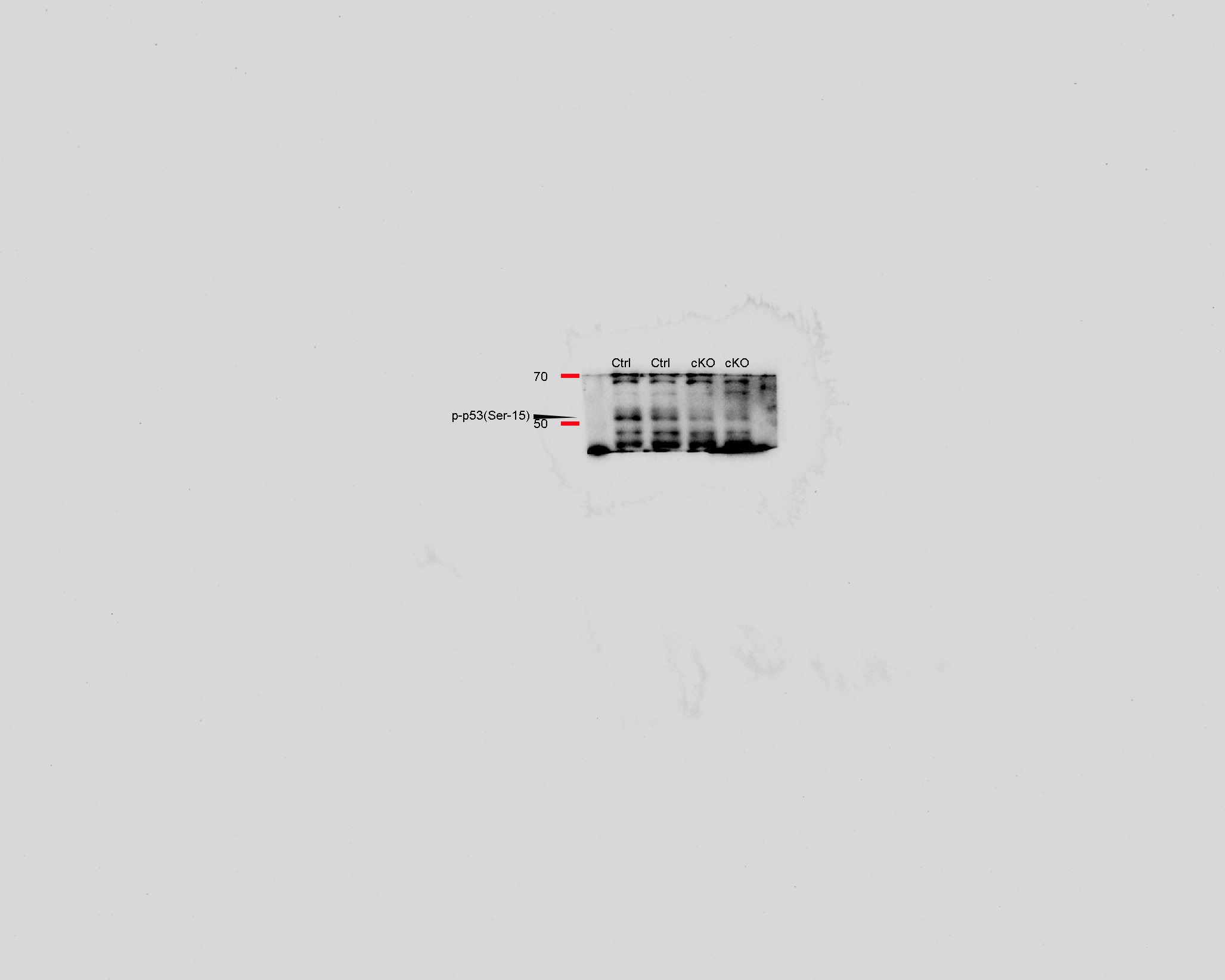

Supplement: Supplementary file 3 — Full length western blots [file 41420_2026_2974_MOESM3_ESM.zip › Fig.8D p-P53.tif]

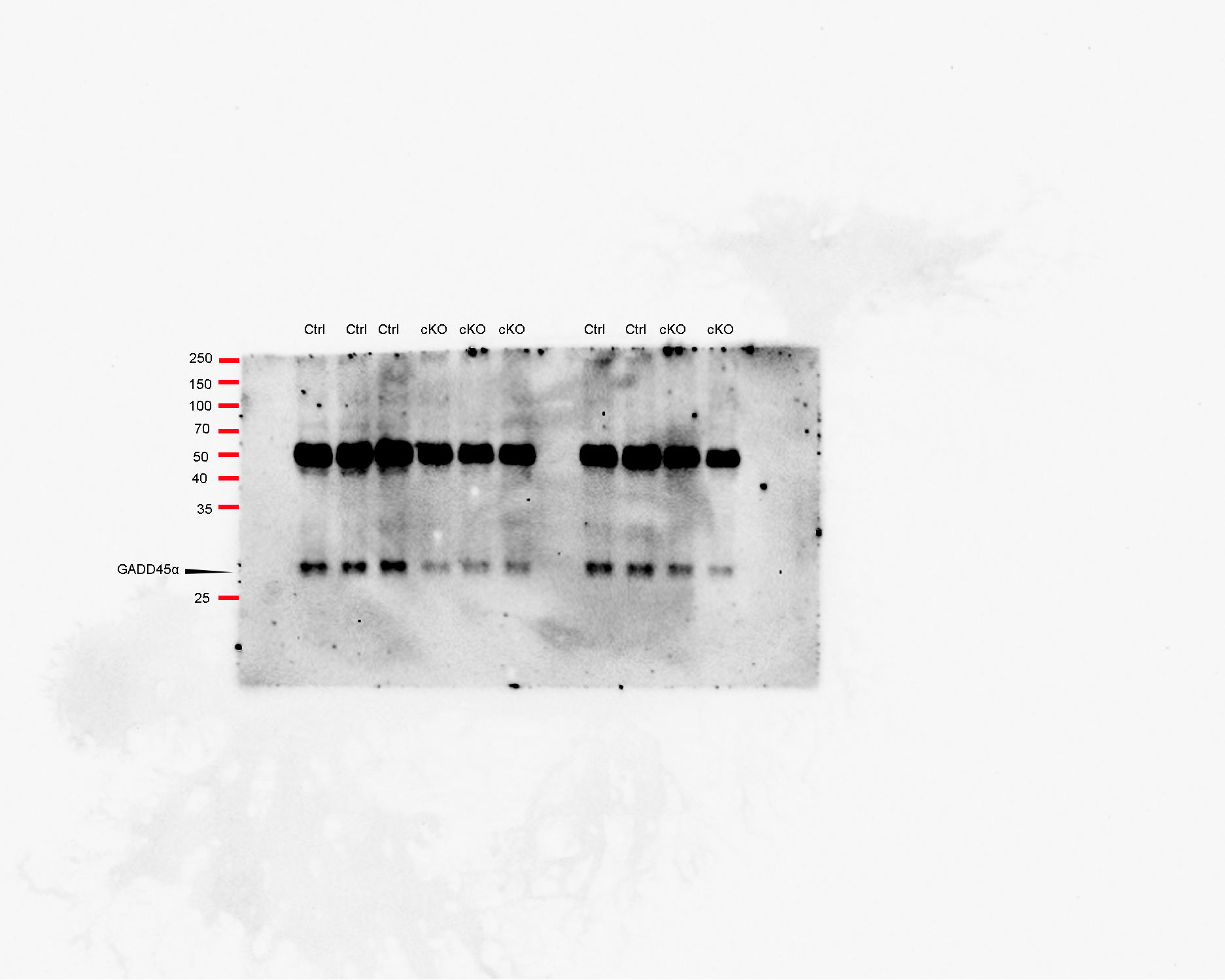

Supplement: Supplementary file 3 — Full length western blots [file 41420_2026_2974_MOESM3_ESM.zip › Fig.8F GADD45α.tif]

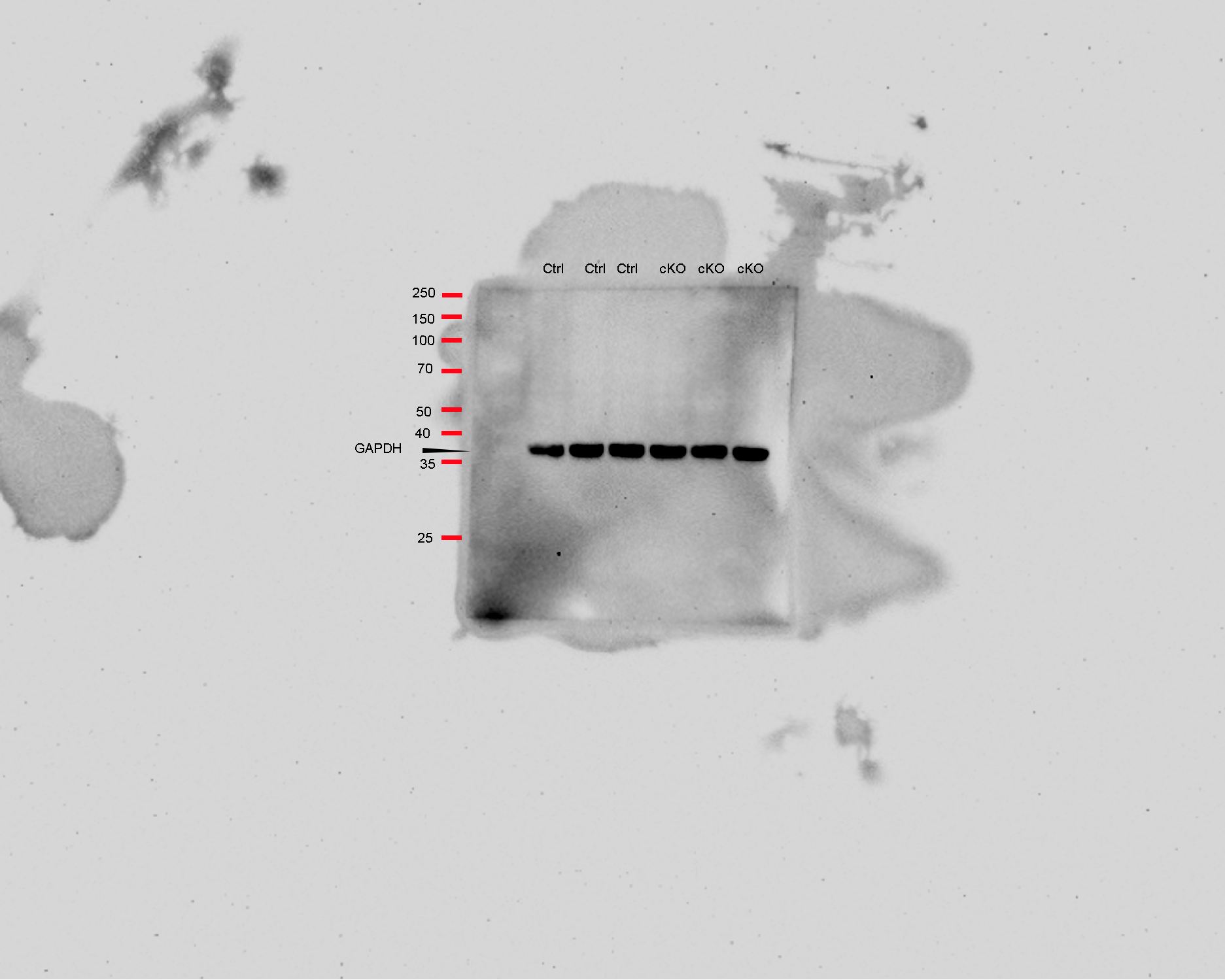

Supplement: Supplementary file 3 — Full length western blots [file 41420_2026_2974_MOESM3_ESM.zip › Fig.8F GAPDH.tif]

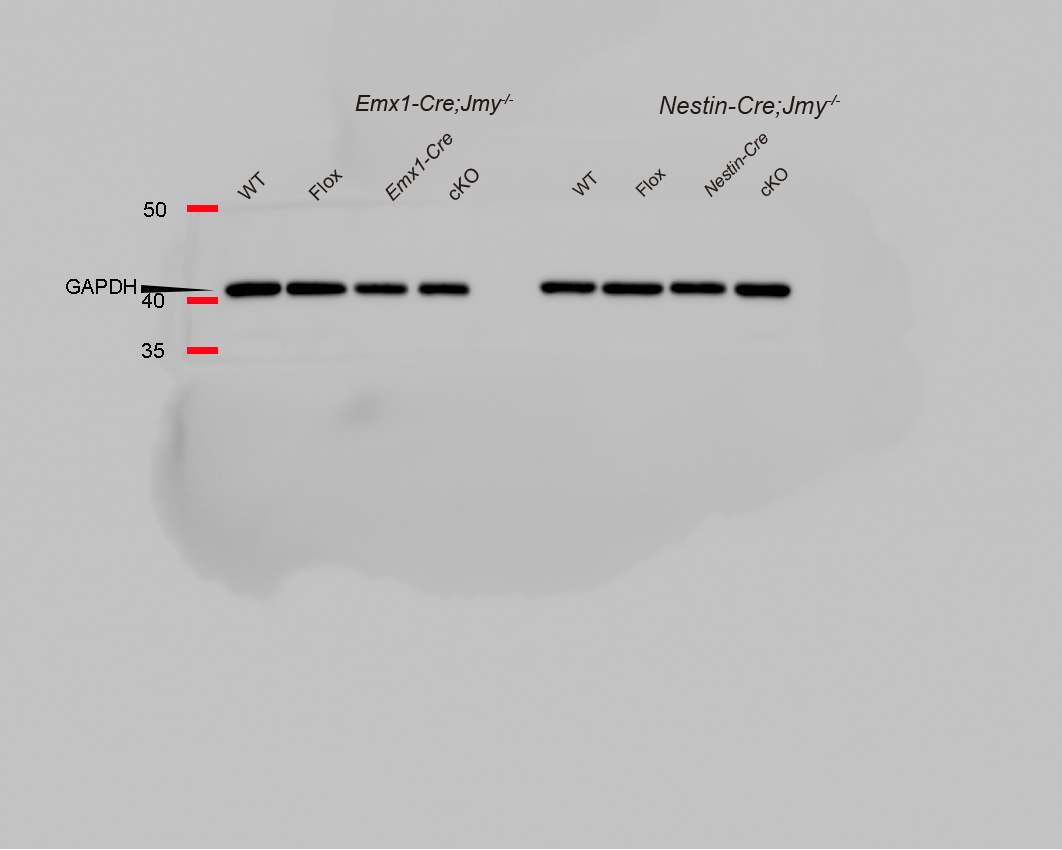

Supplement: Supplementary file 3 — Full length western blots [file 41420_2026_2974_MOESM3_ESM.zip › Supplementary Fig.1D GAPDH(right).tif]

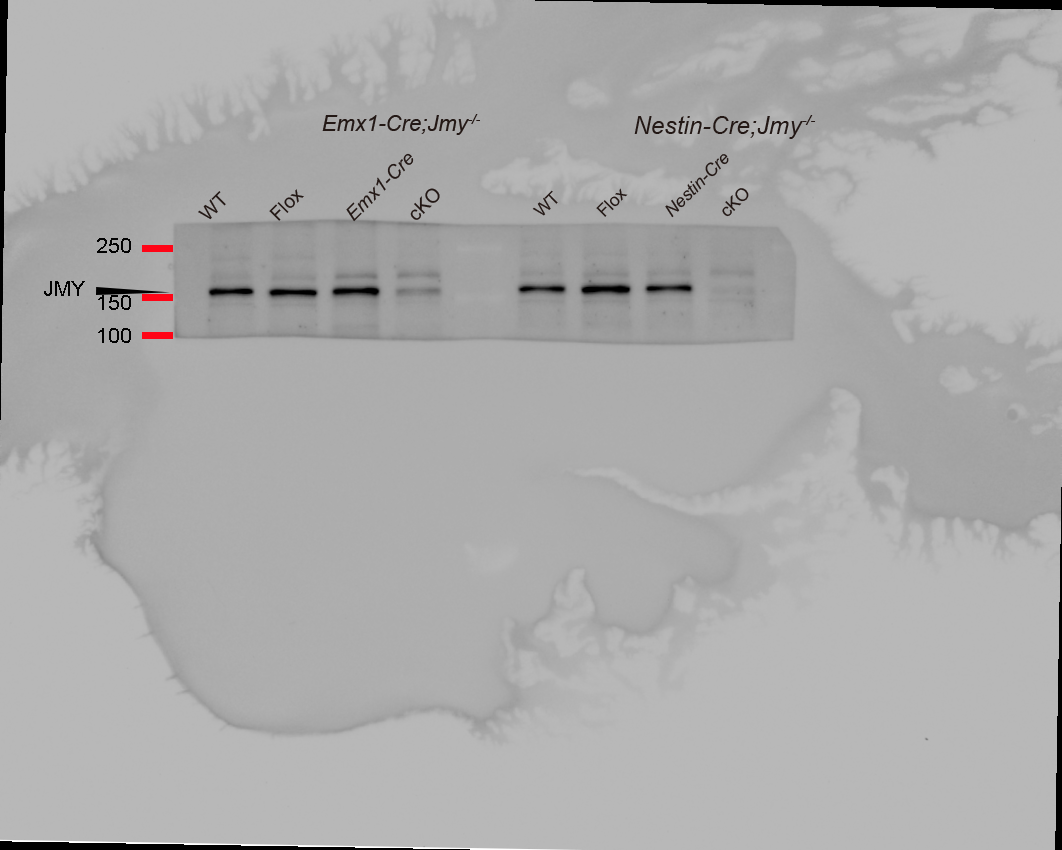

Supplement: Supplementary file 3 — Full length western blots [file 41420_2026_2974_MOESM3_ESM.zip › Supplementary Fig.1D JMY(right).tif]

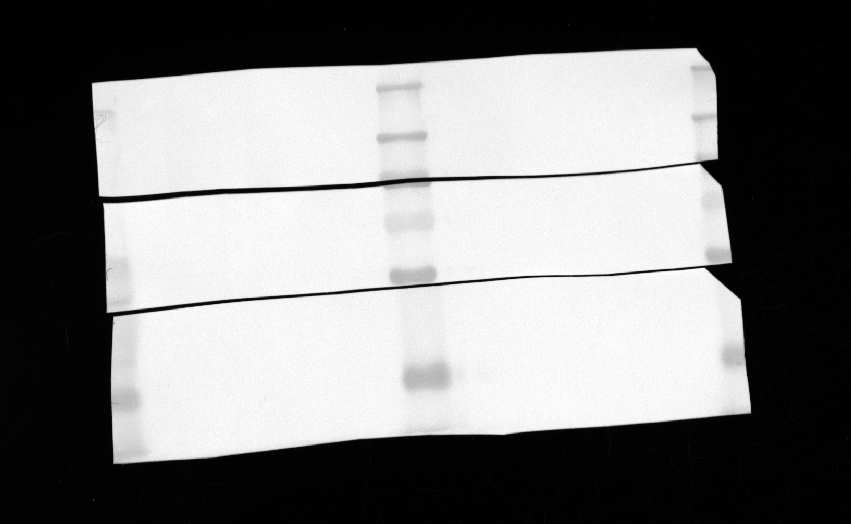

Supplement: Supplementary file 3 — Full length western blots [file 41420_2026_2974_MOESM3_ESM.zip › Supplementary Fig.1D-I Splicing.tif]

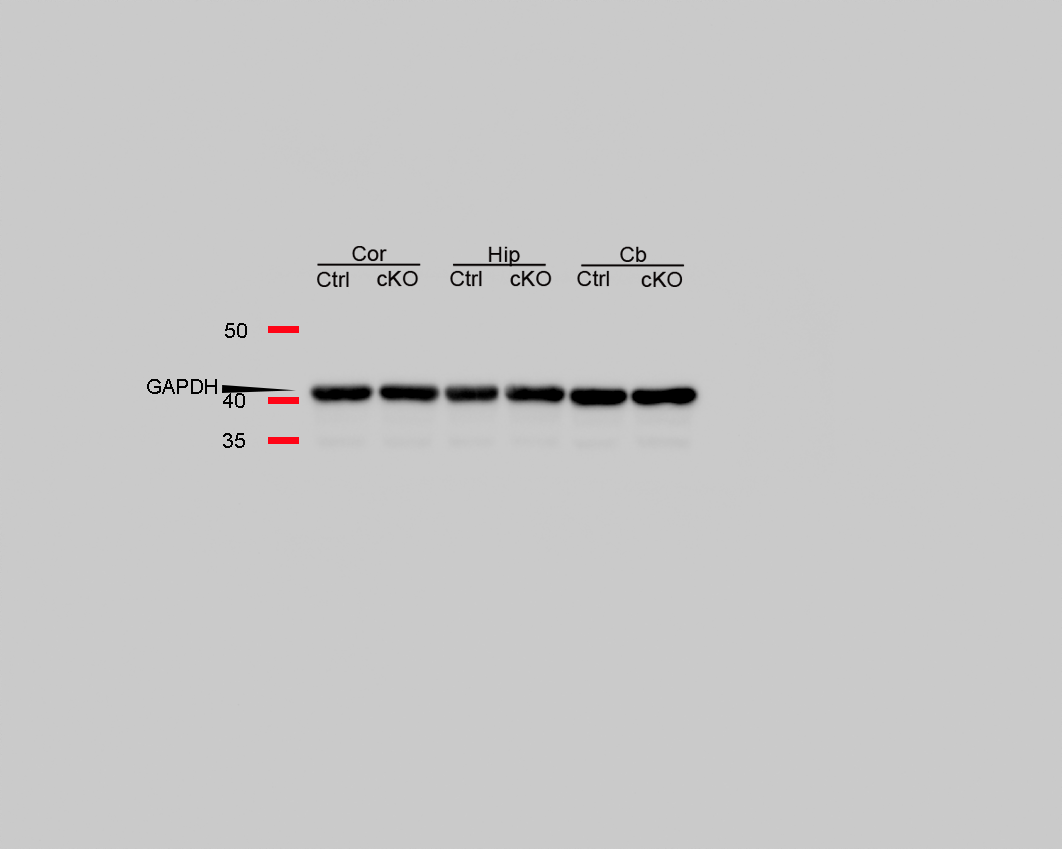

Supplement: Supplementary file 3 — Full length western blots [file 41420_2026_2974_MOESM3_ESM.zip › Supplementary Fig.1I GAPDH.tif]

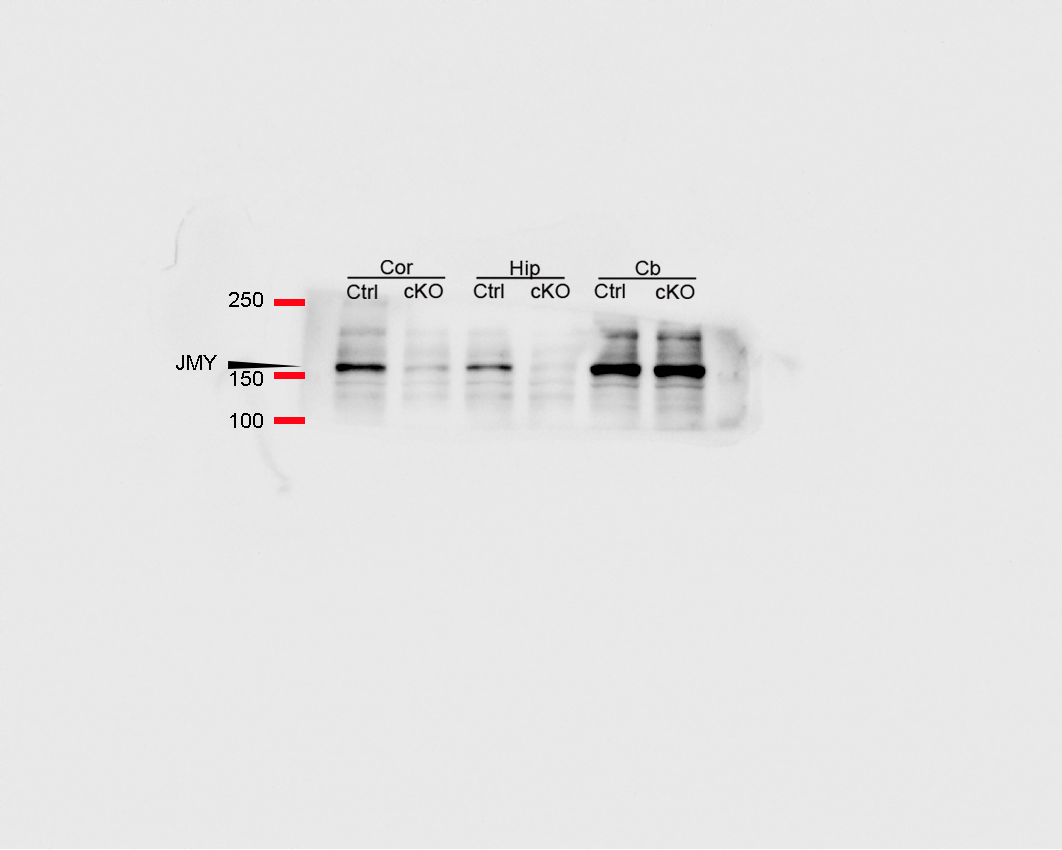

Supplement: Supplementary file 3 — Full length western blots [file 41420_2026_2974_MOESM3_ESM.zip › Supplementary Fig.1I JMY.tif]
